# Coal-Derived Humic Substances: Insight into Chemical Structure Parameters and Biomedical Properties

**DOI:** 10.3390/molecules29071530

**Published:** 2024-03-29

**Authors:** Maria V. Zykova, Kristina A. Bratishko, Evgeny E. Buyko, Lyudmila A. Azarkina, Vladimir V. Ivanov, Dmitrii A. Mihalyov, Evgeniya S. Trofimova, Marina G. Danilets, Anastasia A. Ligacheva, Andrey I. Konstantinov, Alexander A. Ufandeev, Evgenia S. Rabtsevich, Larisa A. Drygunova, Anastasia P. Zima, Sergey R. Bashirov, Elena V. Udut, Mikhail V. Belousov

**Affiliations:** 1 Pharmaceutical Faculty, Siberian State Medical University, 634050 Tomsk, Russia; kr-1295@mail.ru (K.A.B.); buykoevgen@yandex.ru (E.E.B.); ludmila_logvinova@mail.ru (L.A.A.); ivanovvv1953@gmail.com (V.V.I.); diman021999@gmail.com (D.A.M.); trofimova_es@pharmso.ru (E.S.T.); ufandeev@gmail.com (A.A.U.); evgenia882-a@mail.ru (E.S.R.); l_drygunova@mail.ru (L.A.D.); zima.ap@ssmu.ru (A.P.Z.); bars-tomsk@rambler.ru (S.R.B.); evu8@mail.ru (E.V.U.); mvb63@mail.ru (M.V.B.); 2 Goldberg Research Institute of Pharmacology and Regenerative Medicine, 634050 Tomsk, Russia; m.danilets@mail.ru (M.G.D.); vittelli@mail.ru (A.A.L.); 3 Department of Chemistry, Lomonosov Moscow State University, Leninskiye Gory 1-3, 119991 Moscow, Russia; konstant@med.chem.msu.ru; 4 Tomsk State University, 634050 Tomsk, Russia

**Keywords:** humic substances, brown coal, chemical safety, microbiological safety and pharmacological safety, cytoprotectors, antioxidants, immunomodulators, adaptogens

## Abstract

An investigation was carried out on humic substances (HSs) isolated from the coal of the Kansk-Achinsk basin (Krasnoyarsk Territory, Russia). The coal HSs demonstrate the main parameters of molecular structure inherent to this class of natural compounds. An assessment was performed for the chemical, microbiological, and pharmacological safety parameters, as well as the biological efficacy. The HS sample meets the safety requirements in microbiological purity, toxic metals content (lead, cadmium, mercury, arsenic), and radionuclides. The presence of 11 essential elements was determined. The absence of general, systemic toxicity, cytotoxicity, and allergenic properties was demonstrated. The coal HS sample was classified as a Class V hazard (low danger substances). High antioxidant and antiradical activities and immunotropic and cytoprotective properties were identified. The ability of the HS to inhibit hydroxyl radicals and superoxide anion radicals was revealed. Pronounced actoprotective and nootropic activities were also demonstrated in vivo. Intragastric administration of the HS sample resulted in the improvement of physical parameters in mice as assessed by the “swim exhaustion” test. Furthermore, intragastric administration in mice with cholinergic dysfunction led to a higher ability of animals with scopolamine-induced amnesia to form conditioned reflexes. These findings suggest that the studied HS sample is a safe and effective natural substance, making it suitable for use as a dietary bioactive supplement.

## 1. Introduction

The annual production and marketing of dietary supplements made from natural raw materials show a trend of significant growth globally. This trend is primarily driven by the inability of consumers to obtain adequate amounts of biologically active substances through their diet. Consequently, issues pertaining to the quality control and safety of these products are of particular relevance worldwide [1,2]. In accordance with established legislation, the regulation of dietary supplement commerce in the United States is similar to that of food products, meaning that manufacturers are not required to conduct procedures to prove their therapeutic effectiveness, quality, or safety. Such responsibilities are assigned to the Office of Dietary Supplements (ODS), which operates under the National Institutes of Health (NIH). A major tightening of post-marketing pharmacovigilance regulations in the U.S. only occurred after the enactment of the Dietary Supplement and Nonprescription Drug Consumer Protection Act (DSNDCPA) in 2006. Under this act, manufacturers and organizations involved in packaging and marketing dietary supplements are obliged to report adverse side effects to the Food and Drug Administration (FDA) no later than 15 working days after receiving such information.

In the European Union (EU), two laws were gradually developed and adopted that dealt with the regulation of these products [3,4]. The Directive of 1999 allowed manufacturers in EU countries to confirm the safety and efficacy of products by using only literature references concerning well-established medicinal plant-based products. A later directive (EU Directive 2004/24/EC) requires member states to establish a simplified registration procedure for herbal products that do not meet the criteria for medicinal product registration [5]. In Russia, also a legislative, regulatory, and methodological framework has been established to ensure the safe use of dietary supplements. According to this, the responsibility for the quality, safety, effectiveness, reliability, and completeness of information provided about the products lies solely with the manufacturer [6].

It is important to note that legislative requirements globally do not impose any pharmacological safety and efficacy standards for dietary supplements. Requirements are limited to microbiological purity and the content of toxic metals. As for the biological effectiveness of these products, toxicity class, potential allergenic effects on the body, and possible consequences of long-term use—these remain the responsibility of the manufacturers. End consumers can only trust the information provided on the manufacturer’s packaging, as there are no other means to obtain reliable information. Literature references may not always be useful in the context of dietary supplements derived from natural raw materials because the properties of the extracted substances can only be identical if strict adherence to the extraction protocol and the technological regulations for obtaining extracts is maintained. The chemical composition and biological activity of the extracts are directly dependent on variables such as the solvents used, temperature conditions, region of raw material procurement, etc., about which there is a vast amount of literature.

The proper methodology and ethical considerations involved in researching are essential for dietary supplement manufacturers to ensure consumer safety and efficacy. Adhering to good practices demonstrates a commitment to providing robust evidence supporting the quality of a novel substance or natural extract products.

Currently, international markets are seeing an increase in various food products based on humic substances (HSs). However, virtually none of the manufacturers provide any scientific substantiation of their product’s biological safety and efficacy parameters.

From the perspective of food application, HSs are defined as dark-colored organic compounds that can only be derived from natural resources such as peat, brown coal, and sapropel, with a possible presence of HSs in shilajit as well. It should be noted that the synthesis of HSs through artificial means is not feasible, as the humification process occurs naturally over thousands to millions of years. Chemically, HSs are complex assemblies of molecules [7] that incorporate structures with both polymeric and supramolecular characteristics, lacking a strict constancy in chemical composition. They are characterized by a broad range of molecular weights and also exhibit colloidal properties. While HSs from different sources share some molecular parameters, each HS is overall unique in its composition [8,9,10,11,12,13,14]. Therefore, HSs are exceedingly complex natural entities for research and necessitate specific methodological approaches. It is not possible to have a prior assumption about the chemical parameters and biological activity of a particular HS from a given raw material source; thus, each HS must be investigated individually. The literature describes a plethora of pharmacological effects for HSs derived from various raw sources, making these molecules very promising for the discovery and development of novel pharmaceuticals and next-generation food products [9,10,11,12,13,14,15,16,17,18,19,20].

Thus, the aim of this study is to investigate the descriptors of the chemical structure of HSs derived from brown coal and their biomedical properties. This includes evaluating the chemical, microbiological, and pharmacological safety parameters and biological effectiveness of the new substance as an active pharmaceutical ingredient. This is one of the very important aspects in the development of a medical product or food supplement, when a preliminary thorough study of the starting natural raw materials from which the finished product is planned to be created is required.

## 2. Results and Discussion

### 2.1. Physico-Chemical Characterization

**Figure 1:**
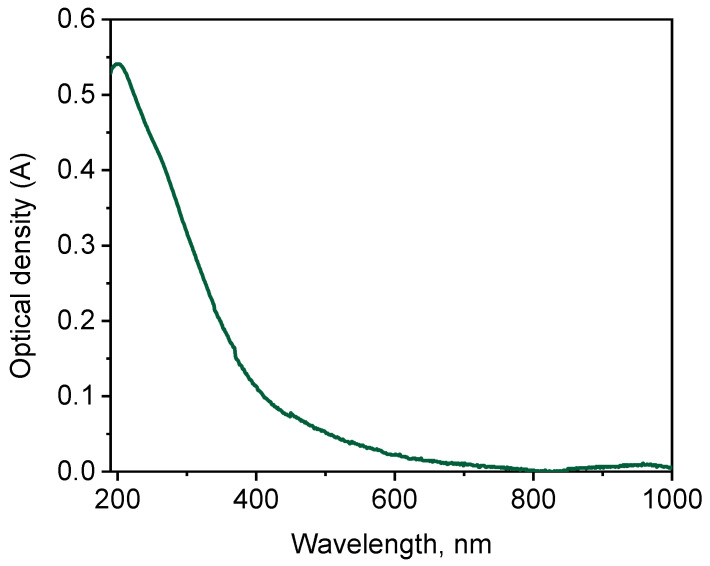
UV-Vis spectrum of the studied HS sample.

The optical properties serve as a critical diagnostic indicator in the examination of HSs [8,21]. The electronic absorption spectrum outline of the studied sample of the HS (Figure 1) manifests as a gentle curve characterized by continuous absorption, with a gradual decrease in optical density as the wavelength increases. The spectral coefficients A465 and A665 are particularly specific; they are in the characteristic range for natural HSs (0.01–0.2). According to E. Velte, the spectral ratio A_465_/A_665_ for the HS’s structure has values between 3 and 5. For the analyzed coal HS sample, the spectral coefficients are 0.0501 ± 0.0071 (A_465_) and 0.0128 ± 0.0018 (A_665_). The A_465_/A_665_ ratio is 3.9260 ± 0.0014, which indicates natural HSs [22].

**Figure 2:**
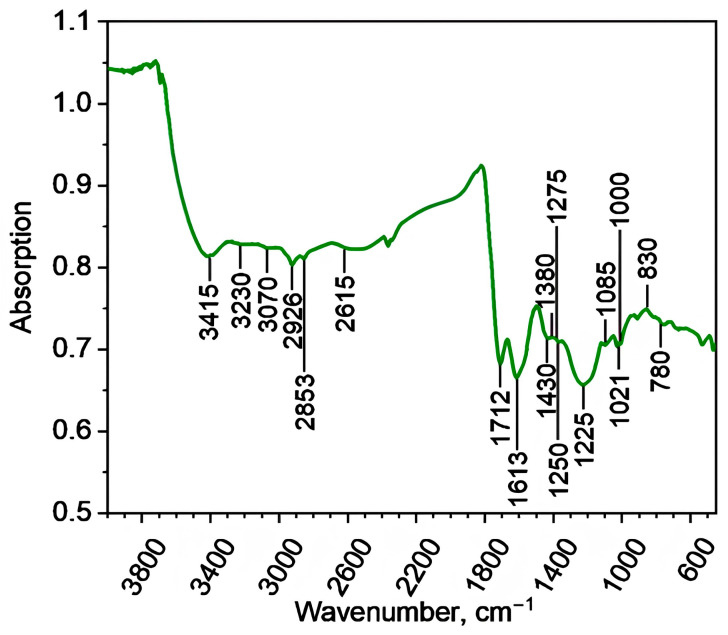
FT-IR spectrum of the studied HS sample.

The infrared (IR) spectrum of the studied coal HS is given in Figure 2 and features characteristic absorption bands [8,23,24,25,26,27,28]. In the IR spectrum, a broad and strong band is observed between 3500 and 3300 cm^−1^ with the most intense absorption at 3415 cm^−1^. This band corresponds to the stretching vibrations of hydroxyl groups (υOH) found in aliphatic and aromatic compounds, primarily linked by hydrogen bonds. 

The decrease in frequency to 3415 cm^−1^ indicates the formation of hydrogen bonds, as free OH groups typically vibrate at 3650–3585 cm^−1^. Additionally, in the range of 3250–3200 cm^−1^, a secondary band with the most intense absorption at 3230 cm^−1^ shows medium intensity absorption attributed to N–H bond vibrations (υNH) in structures like amides and amines. Lastly, weaker bands at 2926 cm^−1^ and 2853 cm^−1^ are observed in the 2930–2920 cm^−1^ and 2860–2850 cm^−1^ regions, respectively, representing stretching vibrations of methyl (–CH_3_) and methylene (–CH_2_–) groups in side hydrocarbon chains of the HS molecule, including aliphatic fragments bounded with aromatic structures. Absorption in the region of 1430 cm^−^¹ is also caused by the bending vibrations of the C–H bond in methylene and methyl groups. But, absorption in 1380 cm^−^¹ region is due to various functional groups in the HS structure. Thus, in this region, asymmetric stretching vibrations of COO^−^ groups can appear in the form of high intensity bands, as well as bending vibrations of the C–H bond in –CH_3_ groups, but of lower intensity.

The changes observed in the 3070 cm^−1^ region are mainly due to vibrations of aromatic =C–H groups in aromatic hydrocarbons with multiple substituents on the ring. In the 2650–2600 cm^−1^ range, there is weak absorption with a peak at 2615 cm^−1^, typical of carboxylic acid dimers. This absorption is related to the stretching vibrations of hydroxyl groups (υOH) involved in strong hydrogen bonds within the dimeric forms formed by hydrogen bonding between –COOH or –COO groups and water. 

The 1725–1700 cm^−1^ range, with a peak at 1712 cm^−1^, corresponds to vibrations of C=O groups found in aldehydes, ketones, carboxylic acids, and their functional derivatives. 

Vibrations in the 1625–1610 cm^−1^ region with the most intense absorption at 1613 cm^−1^ are due to the presence of conjugated C=C (υC=C) and C=O (υC=O) bonds of the aromatic structures, hydrogen bonded carbonyl groups, carboxylate ions, and quinones. However, this band is preferentially associated with vibrations of aromatic C=C bonds.

In 1275–1225 cm^−1^ range, vibrations at 1275 cm^−1^, 1250 cm^−1^, and 1225 cm^−1^ are caused by bonds like C–O and O–H in undissociated carboxylic acids and their functional derivatives, mainly esters. Between 1200 and 1000 cm^−1^, vibrations at 1085 cm^−1^, 1021 cm^−1^, and 1000 cm^−1^ are related to oxygen-containing groups in alcohols, ethers, lactones, esters, and glycosidic bonds in carbohydrates. At 830 cm^−1^, vibrations likely come from C–O bonds in polysaccharide fragments. In the 800–600 cm^−1^ range with a peak at 780 cm^−1^, a weak band is observed, possibly due to bending vibrations of the C–H bond in aromatic rings with two or more unsubstituted hydrogen atoms.

Therefore, it is evident that the IR spectrum is typical for this class of substances, which is a characteristic feature of natural HSs [8,23,24,25,26,27,28].

**Figure 3:**
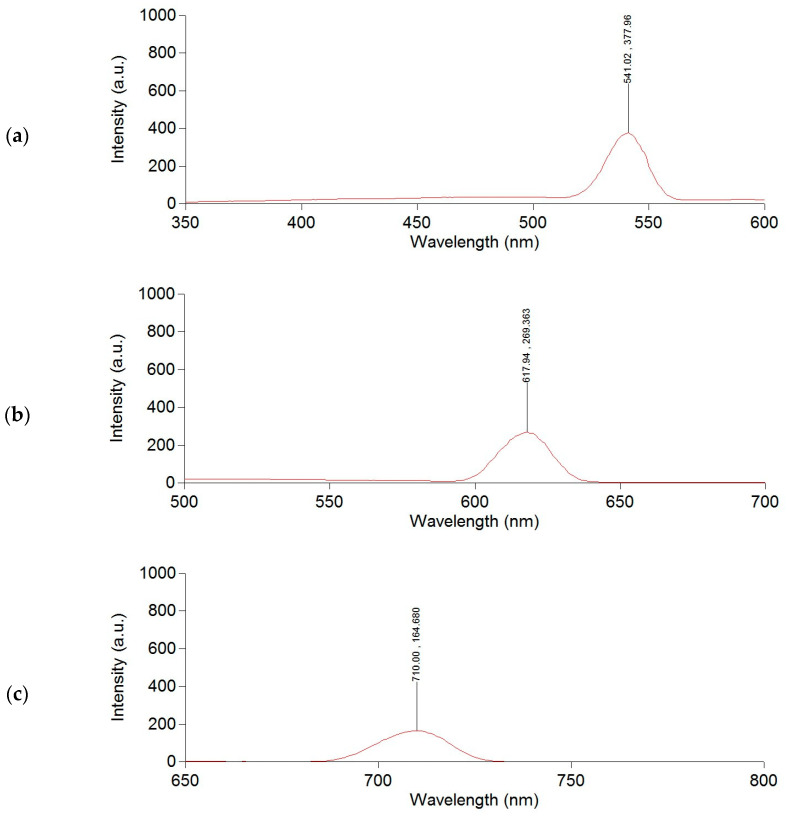
Fluorescence spectra of the studied HS sample at excitation wavelengths of 270 (**a**), 310 (**b**), and 355 nm (**c**).

The fluorescence spectra are presented in Figure 3, appearing as broadened bands in the visible part of the spectrum, with fluorescence maxima centered around 540–550, 610–630, and 705–715 nm at excitation wavelengths of 270, 310, and 355 nm, respectively. Such variability in the position of spectral fluorescence maxima may indicate a high degree of heterogeneity among the fluorophoric groups within the structure of the studied coal HS. Overall, it can be noted that the fluorescence spectra are typical for coal-derived HSs [29].

The data from elemental C, H, N, O analyses are presented in Table 1. The typical content of these elements in natural humic acids falls within the ranges C 46–62 wt%, N 3–6 wt%, H 3–5 wt%, and O 32–38 wt% [24]. However, coal-derived humic acids are usually characterized by significantly lower nitrogen content (2–5 times lower) compared to other sources of HSs [30]. At the same time, it is worth noting that fulvic acids typically have a lower carbon content (36–44 wt%) and a higher oxygen content (45–50 wt%), against a similar content of nitrogen and hydrogen. The studied coal HS sample exhibits certain differences in carbon content (44.91 wt%)—which is lower—and oxygen content (49.48 wt%)—which is higher—reflecting its complex composition, as the sample includes both humic acids and fulvic acids. The calculated values (Table 1) of the H/C ratio suggest that the tested HS sample belongs to the class of hydrocarbons, such as polycyclic aromatic hydrocarbons, which have side aliphatic chains up to 10 carbon atoms in length [31]. The calculated values (Table 1) of the O/C ratio also indicate a high content of oxygen-containing functional groups in the structure of the tested HS [31] Therefore, based on the results presented in Table 1, it can be concluded that the substance under investigation belongs to coal-derived HSs.

**Figure 4:**
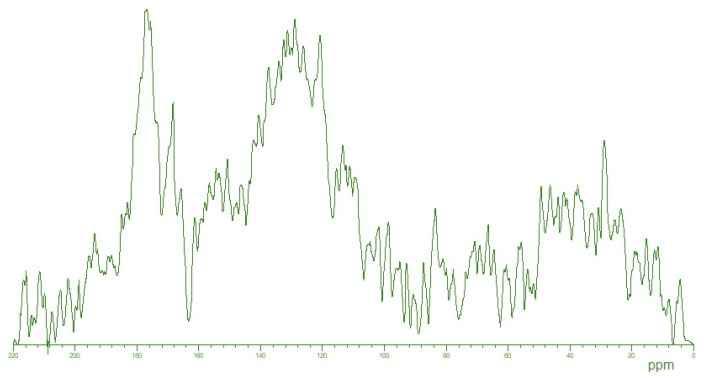
^13^C NMR spectrum of the studied HS sample (signal intensity normalized to the maximum signal of the spectrum).

The ^13^C NMR spectrum is shown in Figure 4. It should be noted that the spectrum exhibits a complex appearance of broad, partially overlapping signals, which is generally typical for natural HSs [30,32]. In the ^13^C NMR spectrum of the tested coal HS sample, we can identify characteristic structural descriptors: from 5 to 48 ppm—the region associated with non-heteroatom-bound carbon, representing aliphatic CH_n_ groups of alkyl fragments; from 48 to 90 ppm—the region of carbon atoms single-bonded to heteroatoms, including aliphatic alkoxy groups, carbohydrate fragments, and amino groups of amino acids; from 90 to 108 ppm—the area of aliphatic carbon bonded to two heteroatoms, often oxygen or nitrogen, commonly observed as the acetal carbons in polysaccharides; from 108 to 145 ppm—the region of unsubstituted and substituted carbon within aromatic fragments; from 145 to 165 ppm—the region of carbon atoms in aromatic fragments substituted with oxygen and nitrogen atoms; from 165 to 187 ppm—the region of carbon within carboxylic, ester, and amide groups; from 187 to 220 ppm—the range for carbon atoms associated with aldehyde, ketone, and quinone groups.

The high-performance size-exclusion liquid chromatography analysis revealed that only 59% of the substance was recovered from the column, suggesting that the studied HS sample is highly hydrophilic. Key molecular weight distribution parameters were calculated. The weight-average molecular weight (Mw) was found to be 30.2 kDa, the number-average molecular weight (Mn) was 3.0 kDa, the peak molecular weight (Mp) was 29.8 kDa, and the polydispersity index (P) as the ratio of Mw/Mn was 10.1, indicating a high polydispersity of the tested HS sample. Overall, the obtained results of the molecular weight distribution suggest that the molecular weights of the tested HS sample are concentrated in the mid-value range, which is typical for naturally occurring HSs [33]. Based on the data obtained, the studied HS sample can be categorized as a highly hydrophilic, polydisperse biopolymer with average molecular weight values (30.2 kDa).

Consequently, the investigation of the chemical structure descriptors of the studied HS sample, based on elemental analysis and IR and ^13^C NMR spectroscopy data, allows us to conclude the significant contribution of aromatic structures to the constitution of the studied HS sample, as well as the high content of oxygen-containing functional groups. Based on elemental analysis and high-performance size-exclusion liquid chromatography, it can be inferred that the studied HS sample has high hydrophilicity and low hydrophobicity. From the data of electronic and fluorescence spectroscopy, it can be suggested that the HS sample exhibits a condensed (non-monomeric) character of the aromatic core and a high degree of polyconjugation.

**Figure 5:**
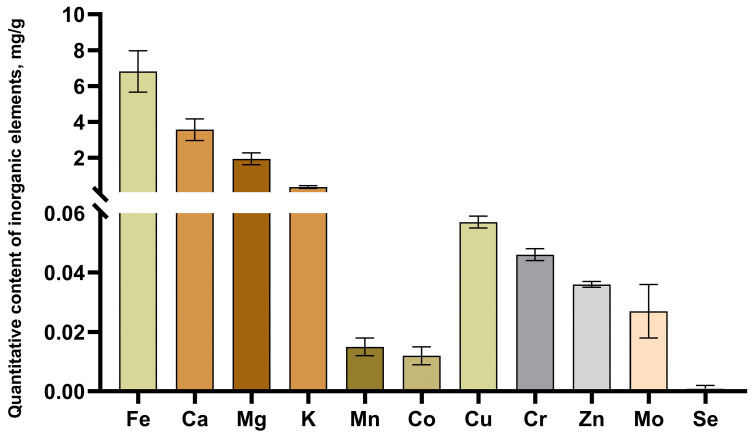
Content of inorganic elements in the composition of the studied HS sample.

Additionally, Inductively Coupled Plasma–Mass Spectrometry (ICP-MS) identified 11 biogenic chemical elements in the studied HS sample (Figure 5). The content of radionuclides was calculated according to General Pharmacopoeia Monograph (GPM) 1.5.3.0001.15 (“Determination of radionuclide content in medicinal plant raw materials and medicinal herbal preparations”). Radionuclide content in medicinal plant raw materials and medicinal plant preparations) in the studied HS sample was not detected, with allowable limits being no more than 400 Bq/kg for Cs-137 and no more than 200 Bq/kg for Sr-90. The levels of heavy metals and arsenic were calculated according to GPM 1.2.2.2.0012.15 (“Heavy metals”) [34,35]. Heavy metals did not exceed the required values (lead 0.0004834 mg/g, which is no more than 6.0 mg/kg; cadmium 0.0000128 mg/g, which is no more than 1.0 mg/kg; mercury 0.0000311 mg/g, which is no more than 0.1 mg/kg; arsenic 0.0000242 mg/g, which is no more than 0.5 mg/kg) [36,37].

### 2.2. Antioxidant Activity Study

The antioxidant activity of natural substances is often one of the primary mechanisms of their pharmacological activity. This is due to the fact that the existence of living organisms, from the simplest to the most complex multicellular structures (with the exception of a small number of obligate anaerobes), is associated with the absorption and utilization of molecular oxygen in the respiratory chain of mitochondria.

From studying the antioxidant activity (AOA) of the HS sample, the ability to reduce the levels of stable free radical’s DPPH and ABTS•^+^ has been demonstrated, comparable to the reference compounds dihydroquercetin and trolox (Table 2).

The studied HS sample demonstrated a high capacity to inhibit free radicals O_2_^−^• (superoxide anion) and HO• (hydroxyl radical) in model systems, comparable to the reference compounds ascorbic acid and mannitol, respectively (Table 2). This activity is likely due to the presence of a high content of phenolic and quinoid groups, as well as semiquinone-type radicals in their structure [9,38,39,40,41]. Apart from quinoid/semiquinoid moieties, phenolic groups oxidized to phenoxyl radicals can also contribute to the antioxidant activity of HSs [42].

The method for measuring total antioxidant activity involves using a stable cation-radical ABTS•+. Antioxidants act by inhibiting this cation-radical through proton donation and electron transfer, which leads to a decrease in the concentration of ABTS•+ and a reduction in the absorption of the solution in the experimental model [43].

The research results indicate that the studied HS sample has a pronounced ability to reduce the concentration of the cation-radical ABTS•^+^ in the model system. The half-maximal inhibitory concentration (IC_50_) for the HS sample was found to be 10.8 ± 0.3 μg/mL, which is comparable to the reference drug Trolox 3.4 ± 0.2 μg/mL (Table 2).

Colorimetry of free radicals, based on the reaction with a 1,1-diphenyl-2-picrylhydrazyl (DPPH) stable chromogenic radical in an alcohol solution, has long been used as a convenient method for analyzing the antioxidant activity of various biological materials (amino acids, vitamins, plant extracts, etc.). The main advantages of this method are high productivity, simplicity of operations, accessibility of required equipment, high sensitivity, and selectivity towards radical scavenging antioxidants [44]. The essence of the method lies in the interaction of DPPH with radical scavenging antioxidants through a consecutive parallel mechanism [45].

The investigated HS sample showed high efficiency in the DPPH radical model, resulting in pronounced anti-radical activity comparable to the reference drug dihydroquercetin (Table 2). A possible mechanism of the HS sample’s anti-radical activity towards ABTS•^+^ and DPPH radicals may be its ability to act as a proton donor due to the presence of a large number of phenolic and carboxyl groups. 

The voltametric method for determining antioxidant activity by registering the cathodic oxygen reduction current is one of the most effective and highly sensitive techniques for measuring the oxygen content in both aqueous and non-aqueous environments [46,47,48].

The investigated HS sample’s antioxidant activity was assessed using the catalytic activity coefficient “K” expressed in µmol/L*min, which characterizes the reducing ability [47,48]. The catalytic antioxidant activity study results are presented in Table 3.

It can be noted that the catalytic activity value for the investigated HS sample was 0.91 µmol/L*min, which is comparable to the value for the reference antioxidant ascorbic acid and exceeds the value for the natural flavonoid dihydroquercetin (0.78 µmol/L*min) by 16.6%. The possible mechanism of the HS sample antioxidant activity may be related to the ability of quinoid groups to participate in the electrochemical reduction process of O_2_ (EO_2_ process):


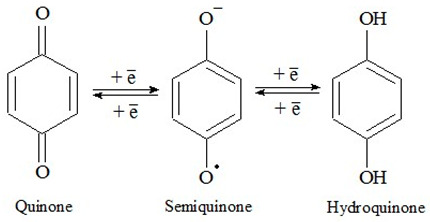


One of the dangerous free radicals in the human organism is O_2_^−^•, which is capable of bypassing endogenous antioxidant defense systems by inactivating certain specific enzymes that protect the body from oxidative damage, particularly glutathione peroxidase, catalase, and others [49]. Additionally, under the action of O_2_^−^•, Fe^3+^ ions bound to ferritin in cells can be released as Fe^2+^ ions, which can generate HO• through the Haber–Weiss reaction. This mechanism is one of the key sources of hydroxyl radical (HO•) formation [50].

The results of determining the antioxidant activity using the nitroblue tetrazolium colorimetric method showed that the HS sample exhibited high effectiveness (IC_50_ of 20.5 ± 1.7 µg/mL) in inhibiting superoxide anion radicals (O_2_^−^•), resulting in pronounced antioxidant activity comparable to the reference drug, ascorbic acid (IC_50_ of 13.3 ± 0.9 µg/mL) (Table 2).

The chelation of metals with a variable valence is one of the most important mechanisms of biologically active substances’ antioxidant activity. When studying the chelating activity, it was shown that the HS sample exhibited a sufficiently high chelating activity (IC_50_ of 25.7 ± 0.5 µg/mL) with the ferrozine-Fe^2+^ complex (Table 2).

HO• radicals are one of the most dangerous reactive oxygen species causing oxidative damage to DNA, proteins, and membrane lipids [51] due to their extremely high reactivity and short lifespan. On the other hand, cells do not produce enzymes to neutralize this particular type of reactive oxygen species [52].

The percentage of inhibition was found to be 93.1 ± 1.2% at 2 mg/mL HSs without EDTA. The level of inhibition can be attributed both to the ability of the HS to interact with HO• and to the ability of the HS to bind iron ions, as shown in the previous trial. Therefore, a parallel study of HO• inhibition was carried out with the addition of EDTA to neutralize the chelating activity of the HS. 

The inhibition decreased to 43.1 ± 1.9% due to the fact that EDTA is a stronger iron ion complexer. A stronger complexon binds iron ions to form a complex capable of generating the HO• radical. Here, the inhibitory effect on malondialdehyde formation is mainly due to the ability of the HS to neutralize the HO• radical.

In the presence of EDTA, the IC_50_ for the HS sample was 2.4 ± 0.2 mg/mL, which is comparable to the antioxidant activity of the classical hydroxyl radical trap, mannitol, which inhibits the formation of malondialdehyde from deoxyribose by 50% at a concentration of 0.9 mg/mL (Table 2). It is probable that the studied HS sample realizes its ability to inhibit HO• through two pathways, one involving direct interaction with HO• and the other through metal chelation.

### 2.3. Cytotoxicity Study

**Figure 6:**
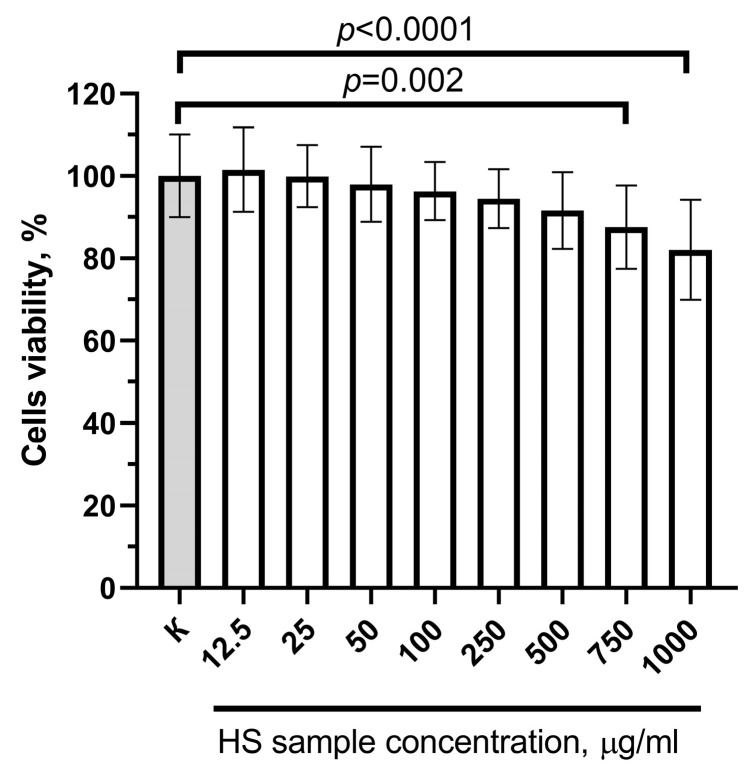
Effect of the studied HS sample on the viability of HepG2 cells after 24 h incubation. Note: for all concentrations (*n* = 18).

The evaluation of the HS sample cytotoxic effects towards the HepG2 cell line using the MTT assay after 24 h of incubation showed that the studied sample suppressed the cell culture’s viability only at the highest concentrations, with a toxic concentration of 750 µg/mL (Figure 6).

The studied HS sample showed an IC_50_ value of 5251 ± 621.7 µg/mL towards the HepG2 cell line, suggesting that it does not exhibit significant cytotoxic effects.

### 2.4. Antioxidant Activity Study In Vitro

**Figure 7:**
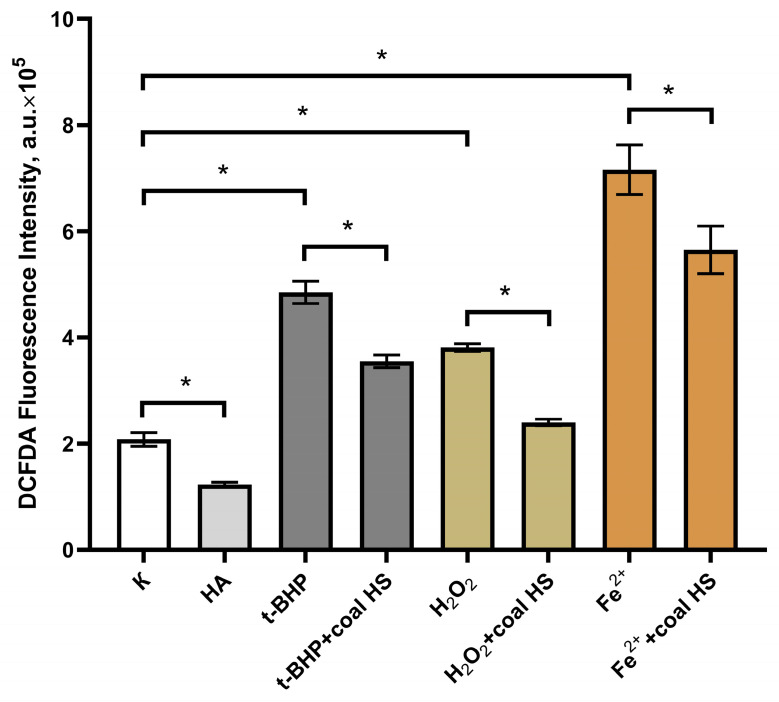
Effect of the studied HS sample on the DCFDA fluorescence intensity in HepG2 cells after addition of pro-oxidants H_2_O_2_, t-BHP, Fe^2+^ ions to the incubation medium (for all *n* = 6). Note: *—the differences are statistically significant, *p* < 0.01.

The antioxidant and antiradical properties of the studied HS sample in cell culture were evaluated based on its ability to reduce the fluorescence intensity of the 2,7-dichlorofluorescein diacetate (DCFDA) probe, following the stimulation of free radical processes after adding oxidants (hydrogen peroxide (H_2_O_2_), tert-butyl hydroperoxide (t-BHP), and Fe^2+^ ions to the HepG2 cell line. The results of the study are presented in Figure 7. The fluorescence intensity of DCFDA in cells following incubation with the HS sample (25 µg/mL) for 24 h was 1.23·10^5^ ± 0.04·10^5^ arbitrary units (a.u.), which was significantly lower compared to the control cells (2.08·10^5^ ± 0.13·10^5^). Therefore, treatment of human hepatocellular carcinoma cells with the HS sample resulted in a reduction in reactive oxygen species levels within the cells (Figure 7).

The fluorescence intensity of cells subjected to oxidants was higher than that of control cells and was 4.85·10^5^ ± 0.21·10^5^; 1.74·10^5^ ± 0.07·10^5^; and 7.16·10^5^ ± 0.47·10^5^ arbitrary units for tert-butyl hydroperoxide, hydrogen peroxide, and Fe^2+^ ions, respectively. Pre-incubation of the cell culture with the HS sample at a concentration of 25 µg/mL for 24 h led to a decrease in the fluorescence intensity of cells after the addition of oxidants (3.55·10^5^ ± 0.12·10^5^; 1.22·10^5^ ± 0.06·10^5^; and 5.65·10^5^ ± 0.45·10^5^ arbitrary units for tert-butyl hydroperoxide, hydrogen peroxide, and Fe^2+^ ions, respectively) (*p* < 0.01 for all values).

Thus, pre-incubation of human hepatocellular carcinoma cells HepG2 with the HS sample (25 µg/mL) reduces the levels of intracellular reactive oxygen species after the induction of oxidative stress by oxidants (tert-butyl hydroperoxide, hydrogen peroxide, and Fe^2+^ ions). 

Considering that chronic diseases, such as cancer, cardiovascular disease, and neurodegenerative disorders, are associated with oxidative stress [53], and that the molecular mechanisms of oxidative stress in chronic diseases are realized at the cellular level [54], our in vitro results indicate the high cytoprotective potential of the studied HS sample. 

Numerous studies have shown that natural antioxidants have the ability to scavenge free radicals, thereby contributing significantly to the management of oxidative pathologies [55]. Dietary antioxidants, derived from natural sources, are of particular interest because of their potential health benefits, as they can provide protection against diseases that result from oxidative stress [55,56,57].

### 2.5. Immunopharmacological Study

**Figure 8:**
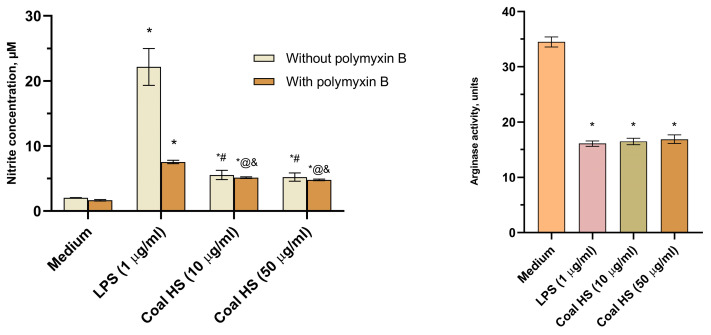
Effect of the studied HS sample on the activity of nitrogen oxide (II) synthase and arginase in peritoneal macrophages of intact C57BL/6 mice. *—Differences from the medium are significant (*p* < 0.05); #—Differences when incubated with Lipopolysaccharide (LPS) control without polymyxin B are significant (*p* < 0.05); @—Differences when incubated with the HS sample without polymyxin B are significant (*p* < 0.05); &—Differences when incubated with LPS in the presence of polymyxin B are significant (*p* < 0.05).

The screening results for the immunopharmacological properties related to the influence of the studied HS sample on macrophage polarization are shown in Figure 8. Macrophages are immunocompetent cells of the body that interact with antigens and present them to the immune system (Th0); depending on the type of antigen, they begin to produce specific cytokines, which cause the polarization of Th0 into Th1 or Th2. Therefore, macrophages are a crucial element in the formation of the immune response. They are responsible for initiating cellular or humoral immune responses. Assessing the impact of HSs on the balance of nitrogen oxide (II) and arginine, it was noted that there is an enhancement of the Th1 type of immune response, resulting in the formation of effector cells to combat intracellular pathogens (viruses and intracellular bacteria) such as cytotoxic T lymphocytes, which also provide anti-tumor immunity [58]. Accordingly, the investigated coal HS sample exhibits immunotropic action as it polarizes macrophages towards the classical M1 type (shifting arginine metabolism towards the activation of nitric oxide synthase), contributing to the formation and enhancement of the Th1 type immune response. The most effective concentration was found to be 10 µg/mL.

M1 macrophages are recruited immediately after tissue injury and participate in the initial response to infectious processes [59]. They intensify local inflammation by producing a large number of pro-inflammatory cytokines, including TNF-α (tumor necrosis factor-alpha), IL-1β (interleukin-1 beta), IL-6 (interleukin-6), and IL-12 (interleukin-12) [60]. The presence of these cytokines in the microenvironment influences the process of polarization of Th0 into Th1 [61]. Th1 cells are also characterized by the production of pro-inflammatory cytokines (IFN-γ (interferon gamma), IL-2 (interleukin-2), TNF-α (tumor necrosis factor-alpha)), and they mediate cellular immune responses aimed at the elimination of intracellular viral and bacterial infections through the activation of pro-inflammatory macrophages and cytotoxic CD8+ T-cells [62]. Thus, the synergy and interaction of immune cells towards a specific type of immune response, at a particular point in time during a disease or infection, is significantly regulated by the combination of cytokines they produce.

The study of the influence of the tested HS sample on the production of pro-inflammatory cytokines by mouse peritoneal macrophages and lymphocytes was conducted based on this premise. Lipopolysaccharide (LPS) was used as a standard macrophage activator, and to stimulate the pool of T-lymphocytes, mouse splenocytes were treated with the T-cell mitogen Concanavalin A (ConA).

**Figure 9:**
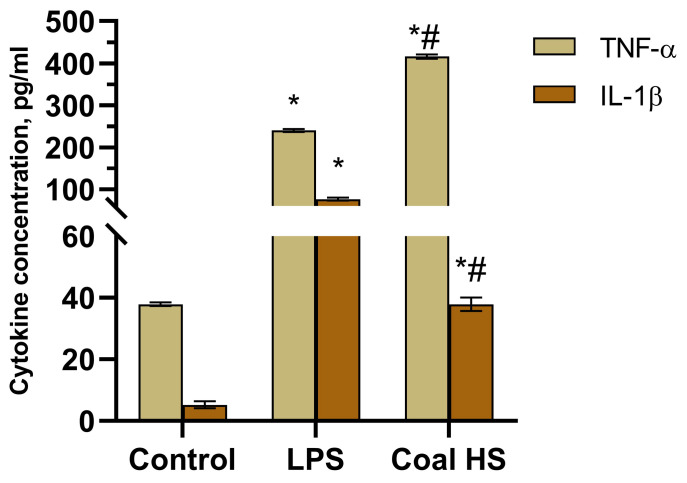
Influence of the studied HS sample on the spontaneous production of TNF-ɑ and IL-1β by peritoneal macrophages of C57BL/6 mice (*n* = 4, mean ± SD); *—*p* < 0.05 in comparison with control, #—*p* < 0.05 in comparison with Lipopolysaccharide (LPS).

As a result, it was determined (Figure 9) that the addition of the coal HS sample to the culture of mouse peritoneal macrophages significantly enhanced the spontaneous production of key pro-inflammatory cytokines TNF-ɑ and IL-1β by 10.9 and 7.2 times, respectively, compared to the intact cells (control). LPS also increased the production of these cytokines by macrophages.

**Figure 10:**
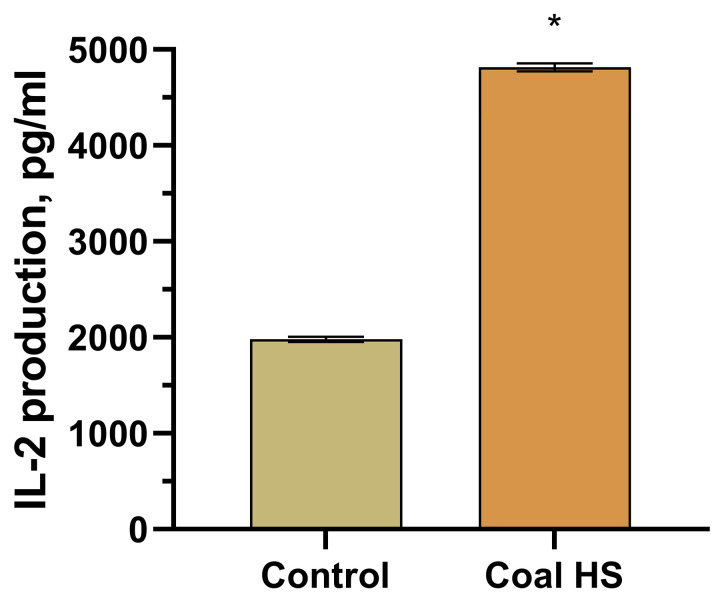
Effect of the studied HS sample on mitogen-stimulated production of IL-2 by splenocytes of C57BL/6 mice (*n* = 4, mean ± SD); *—*p* < 0.05 compared to control. The spontaneous production of IL-2 by mouse splenocytes was 16.25 ± 2.00 pg/mL.

Incubation of Concanavalin A (ConA)-stimulated splenocytes with coal HSs led to a 2.4-fold increase in their production of the pro-inflammatory cytokine IL-2 compared to the control (Figure 10).

Consequently, the studied HS sample induces an increase in the production of pro-inflammatory cytokines by mouse macrophages and splenocytes, confirming the ability of these substances to enhance the Th1 type of immune response. This finding makes the tested HS sample promising for the development of effective immunotropic drugs to correct pathologies associated with Th1-dependent immune response deficiencies, such as chronic, lingering, and recurrent infectious diseases, as well as oncological conditions [63,64,65].

### 2.6. Investigation of Biological Activity In Vivo

#### 2.6.1. Actoprotective Activity

The average swimming time of animals in the control group was 19.5 ± 3.4 min. The administration of the studied HS sample increased the swimming time of the animals to 37.5 ± 9.9 min in the “Meldonium” group and 34.7 ± 4.6 min in the “HS” group (Table 4). The average swimming time of animals after a 10-day course of administration of Meldonium at a dose of 200 mg/kg and the HS sample at a dose of 500 mg/kg was comparable. However, the course administration of the HS sample at a dose of 500 mg/kg significantly increased the swimming time of the mice (*p* = 0.04). The variance in the swimming time parameter in the “Meldonium” group was high; therefore, the increase in the swimming time of animals in this group was not statistically significant (*p* > 0.05).

The administration of the reference drug Meldonium at a dose of 200 mg/kg and the HS sample at a dose of 500 mg/kg resulted in a statistically significant decrease in the concentration of lactate in the serum of animals (Table 5) (*p* < 0.05).

#### 2.6.2. Nootropic Activity

Intraperitoneal administration of scopolamine at a dose of 1 mg/kg 20 min before the training sessions leads to pronounced cholinergic dysfunction in the central nervous system (Table 6), as evidenced by a statistically significant decrease in the conditioned reflex of passive avoidance (CRPA) in mice (*p* = 0.005). Single intragastric administration of the studied HS sample to mice with cholinergic dysfunction resulted in a statistically significant increase in the time spent by the animals on the platform compared to the amnestic control group (*p* < 0.05).

The studied HS sample at a dose of 500 mg/kg facilitates the development of the CRPA in mice in the presence of cholinergic dysfunction induced by the administration of scopolamine at a dose of 1 mg/kg.

A 10-day course of intragastric administration of the HS sample at a dose of 500 mg/kg led to an improvement in the physical performance of mice in the “swimming exhaustion” test. The swimming time of animals receiving the studied sample increased by 77.9% compared to the control group. Additionally, the actoprotective activity of the HS sample was not inferior to that of Meldonium (*p* = 0.39). It is known that the fatigue of peripheral muscles caused by physical exertion is primarily due to lactate accumulation, resulting from anaerobic glycolysis in the muscles [66]. The observed increase in the swimming time of animals following the course administration of the HS sample at a dose of 500 mg/kg may be due to a decrease in the level of lactate in the blood serum of the animals.

A single intragastric administration of the studied HS sample at a dose of 500 mg/kg leads to a restoration of the animals’ ability to develop conditioned reflexes at a control level induced by scopolamine-induced amnesia (*p* = 0.01). The amnestic effect of scopolamine is realized through the induction of cholinergic dysfunction in the central nervous system [67,68]. This allows us to hypothesize that the nootropic effect of HSs is realized through an impact on the cholinergic system.

### 2.7. Toxicity Study

#### 2.7.1. Acute Toxicity

A single intragastric administration of the studied HS sample at a dose of 2000 mg/kg did not lead to the death of animals on the day of administration or during the subsequent 14 days of observation. No clinical signs of health impairment were recorded. The effect of single intragastric administration of the HS sample at a dose of 2000 mg/kg on the dynamics of body weight in animals was also not observed (Table S1).

Upon necropsy of the animals on the 15th day, no macroscopic signs indicating damage to internal organs were found.

A single intragastric administration of the HS sample at a dose of 2000 mg/kg to rats did not lead to their death and did not exert a toxic effect.

#### 2.7.2. Toxicity Study during Multiple Administrations

No clinical signs of health disturbance were observed in rats following a 28-day course of intragastric administration of the studied HS sample at a dose of 1000 mg/kg. The dynamics of body weight in the animals were similar to those in the control group (Table S2). There were no changes in food consumption either (Tables S3 and S4).

The hematological (Table S5) and serum biochemical (Table S6) parameters in rats following the 28-day course of intragastric administration of the studied HS sample at a dose of 1000 mg/kg did not differ from the control values. The kidney metabolic parameters also remained unchanged (Table S7).

The absolute and relative organ weights after the 28-day course of intragastric administration of the HS sample at a dose of 1000 mg/kg did not differ from the control values (Tables S8 and S9). Furthermore, no pathological changes in the organs and tissues were observed, both macroscopically and histologically.

#### 2.7.3. Allergenicity Study

During the assessment of the sensitizing effect of the studied HS sample through dermal applications in guinea pigs, no skin reaction at the application site was observed after 10 and 20 applications (Table S10).

## 3. Materials and Methods

### 3.1. Coal Humic Substance (HS) Sample Preparation

For the analysis of chemical structure descriptors and biomedical properties, the HS sample was derived from lignite coal from the Kansk-Achinsk coal basin (Krasnoyarsk region, Russia). The HS sample was obtained using an extraction method from lignite coal with a 1% aqueous Na_2_CO_3_ solution, involving dispersing operations and cavitation at a temperature of 60 °C and a rotor speed of 3000 rpm. The resulting extract was centrifuged at 4000 rpm and filtered using a noutch-filter, followed by evaporation using a rotary evaporator and drying to an air-dry state at temperatures ranging from 40 to 60 °C. This extraction method enabled the production of a complex composed of humic and fulvic acids. The extracted coal HS sample appeared as an amorphous dark-colored powder. This substance is readily soluble in water as it is a sodium salt.

To carry out research using physicochemical analysis methods, the HS sample was previously desalted by the dialysis method using a nitrocellulose membrane with a pore diameter of 1 kDa and Milli-Q deionized water [69]. To study the pharmacological activity, the HS sample in a water-soluble form of sodium salt was used.

### 3.2. Microbiological Purity

Microbiological purity was determined in accordance with the GPM 1.2.4.0002.15 (“Microbiological Purity”) [70]. In brief, the testing includes methods for sample preparation, collection of samples for analysis, quantitative determination of viable microorganisms and detection and identification of specific bacterial species (aerobic microorganisms, yeast and mold fungi, enterobacteria, *Escherichia coli*, *Salmonella* spp., *Staphylococcus aureus*) whose presence is unacceptable or limited in substances according to the assigned category of microbiological purity, as well as the necessary culture media, solutions, and reagents for conducting the tests. The standard incubation temperature for bacterial cultures on nutrient media is (32.5 ± 2.5) °C and for fungi is (22.5 ± 2.5) °C.

For the sample of HS extracted from coal, the category of microbiological purity 3.2 “Natural origin substances (plant, animal, or mineral) for the production of non-sterile medicinal products” has been established.

### 3.3. Inorganic Elemental Composition by ICP-MS Method

The inorganic elemental composition was determined using an inductively coupled–plasma mass spectrometry (ICP-MS) method with an Agilent 7500cx quadrupole mass spectrometer (Agilent Technologies, Palo Alto, CA, USA). To prepare the samples for ICP-MS analysis, the HS sample was ashed at a temperature of 500 °C for 2 h. The resulting ash residues were then dissolved. The dissolution process involved the use of pre-purified concentrated nitric acid (ultrapure grade, 70%), hydrogen peroxide (30%), and the Milestone Start D microwave digestion system (200 °C, 700 W). Subsequently, the samples were dried at approximately 100–110 °C until a state of wet ash was reached and then quantitatively transferred into disposable 50 mL polypropylene tubes using a background solution of 15% nitric acid. A blank sample was prepared in parallel with the test samples. Prior to analysis, an internal standard solution of indium was added to each tube containing the samples and the blank. Thereafter, all samples were diluted to a consistent volume. The calculation of the final results included accounting for the dilution factor, the internal standard, and the blank sample.

### 3.4. Quantification of Heavy Metals and Arsenic Content

The quantification of heavy metals and arsenic in the HS sample was carried out in accordance with the GPM 1.5.3.0009.15 (“Determination of Heavy Metals and Arsenic in Medicinal Plant Raw Material and Herbal Medicinal Products”) [70]. Additionally, the measurement of radionuclide content in the HS sample was conducted following the GPM 1.5.3.0001.15 (“Determination of Radionuclides in Medicinal Plant Raw Material and Herbal Medicinal Products”) [70].

### 3.5. Physico-Chemical Analysis

#### 3.5.1. Electronic Spectroscopy

The electronic absorption spectra of 0.001% aqueous solutions of the HS sample were recorded using a Unico 2800 spectrophotometer (United Products & Instruments, Inc., NJ, USA) within the wavelength range of λ = 190–800 nm using a 1 cm quartz cuvette. In the obtained spectra, the absorption maxima were identified, extinction coefficients (E 0.001%, 1 cm) at λ = 465 nm (A_465_) and λ = 665 nm (A_665_) were determined, and the ratio of absorption at the wavelengths of 465 and 665 nm was calculated (the coloration index was according to E. Welte (Q_4/6_)) [8,21].

#### 3.5.2. Infrared Spectroscopy

Infrared (IR) spectra were recorded on an FSM 1201 Fourier-transform infrared (FTIR) spectrometer (Infraspect Ltd., Saint Petersburg, Russia) in KBr pellets (with a ratio of 1:100, sample to KBr respectively), in the range of wave numbers from 500 to 4000 cm^−1^. A sample was weighed with an accuracy of 0.0010 g. Absorption maxima were identified in the obtained spectra. 

#### 3.5.3. Fluorescence Spectroscopy

For the investigation of the fluorescent properties, a 0.0004% solution of the HS sample was prepared in phosphate buffer (pH = 6.86). Fluorescence measurements were conducted using a Cary Eclipse spectrophotometer (Agilent Technologies, Palo Alto, CA, USA) with a 1 nm step at excitation wavelengths of λ_ex_ = 270, 310, and 355 nm [29]. The maximum fluorescence wavelengths (λ_max_) were established for λ_ex_ = 270, 310, and 355 nm as the positions of the fluorescence maxima.

#### 3.5.4. Elemental (C, H, N, O) Analysis

Elemental composition analysis of the HS sample was carried out using the automatic semi-microanalysis method on a Carlo Erba Strumentazione Model 1301 automatic CHN analyzer (Carlo Erba Strumentazione, Milan, Italy) for ash-free and anhydrous samples, while the oxygen content was determined by difference. The mass fractions of elements (wt%) were found using calibration curves, which were constructed using the Sulphanilamide reference standard material. Following the widely recognized method, the atomic fractions of elements (atom%) and atomic ratios were calculated [30,31].

#### 3.5.5. ^13^C-NMR Spectroscopy

The 43 mg weight of the coal HS sample under the study was moistened with three drops of 40% NaOH and dissolved in 0.6 mL of 0.3 M NaOD/D_2_O solution (Sigma Aldrich, St. Louis, MO, USA, isotope purity 99+ atomic%). The solution prepared was put into an ultrasound bath for 30 min, followed by centrifugation at 10,000 rpm for 5 min. The solution-state HS sample prepared was separated from the precipitate and put into a 5-mm NMR tube. Quantitative ^13^C NMR spectra were recorded on a Bruker Avance 400 NMR spectrometer operating at 100 MHz ^13^C frequency with the use of a CPMG pulse sequence. The acquisition time was 0.2 s. For suppressing the nuclear Overhauser effect, the broadband decoupling from protons was switched on for free induction decay (FID) acquisition and switched off for the relaxation delay time (INVGATE pulse technique). The duration of relaxation delay was set to 7.8 s on the basis of [32,71]. The total number of scans was 4968, which took about 12 h. To improve the signal-to-noise ratio, the FID signal acquired was multiplied by an exponentially decaying function with a line broadening parameter (lb) of 100 Hz. For exponential line broadening, Fourier transformation, spectrum phasing, and baseline correction, the MestReC software 4.56 was used.

#### 3.5.6. Size-Exclusion High-Performance Liquid Chromatography

The study of the molecular weight distribution (MWD) of the HS sample was carried out using high-performance size-exclusion liquid chromatography. A 0.03 M phosphate buffer at pH 6.8, made with MilliQ filtered distilled water, was used as the mobile phase for the size-exclusion chromatography analysis. Gel chromatographic fractionation was performed using an Abimed chromatography system, which included a high-performance size-exclusion liquid chromatography pump, an autosampler, a glass chromatographic column (15 mm diameter, 25 cm length), a UV spectrophotometric detector, an A/D converter card for analytical signal recording, and a computer for data acquisition. The column was packed with “Toyopearl” TSK HW-55S gel (Tosoh Bioscience GmbH, Griesheim, Germany) with a fractionation range of 1–200 kDa for dextran standards. The chromatographic column was calibrated using sodium polystyrenesulfonates (PSS Polymer Standards Service GmbH, Mainz, Germany) with molecular weights of 45,100, 33,500, 15,800, and 10,200 Da, with polydispersity not exceeding 1.2.

To prepare experimental solutions, an accurate weigh-in of the tested sample (3.5 mg) was dissolved in 25 mL of the eluent, first moistening the substance with one drop of 1 M NaOH. A 1 mL sample was taken automatically. The analytical signal was spectrophotometrically recorded at λ = 254 nm, with the detector sensitivity set at 0.02 a.u., and a flow rate of 1 mL/min for the eluent.

For the analysis of the size-exclusion chromatograms, the original “Geltreat” software developed by A.V. Kudryavtsev was used [33] and calibration curves based on the sodium polystyrenesulfonate standards were calculated, which were then used to determine the molecular weight characteristics of the HS sample. To determine the completeness of the HS sample elution from the column and the amount of substance adsorbed on the gel, off-column quantitation was performed. Chromatograms were presented in terms of the distribution coefficient (Kd) units.

### 3.6. Cytotoxicity Study

The cytotoxic effect of the HS sample on human hepatocellular carcinoma cells, HepG2, was investigated in vitro using the colorimetric assay with an MTT test as described [72]. The method is based on the ability of the dehydrogenases in the mitochondria of living cells to reduce the water-soluble 3-(4,5-dimethylthiazole-2-yl)-2,5-diphenyl tetrazolium bromide (MTT) into formazan crystals, which are soluble in an organic solvent, dimethyl sulfoxide (DMSO). The absorption of the resultant solution, measured at 540 nm, is proportional to the number of metabolically active viable cells. Prior to experimentation, the HepG2 cell line was cultured under standard conditions. The viability of cells after incubation with the studied HS sample for 24 h at concentrations up to 1000 µg/mL was calculated relative to the viability of control cells and presented as a percentage of viable cells.

### 3.7. Antioxidant Capacity Study

#### 3.7.1. Total Antioxidant Capacity Study

The interaction between the HS sample and the stable cationic free radical ABTS•^+^ (2,2′-azino-bis(3-ethylbenzothiazoline-6-sulfonic acid) diammonium salt) was assessed by evaluating the reduction in its concentration in the reaction medium in the presence of various concentrations of the HS sample [41,73]. Absorption was measured using an SF-2000 spectrophotometer from OKB Spectr (Russia) at a wavelength of 734 nm against a reference solution containing 50 µL of the HS solution at relevant concentrations and 450 µL of purified water. Antioxidant activity was evaluated based on the IC_50_ value—the concentration of HSs at which the concentration of the ABTS•^+^ cation-radical in the given model system was reduced by half. Trolox (Acros Organics, Geel, Belgium), a water-soluble analog of tocopherol, was used as a positive control. The antioxidant activity (AOA) of the HS was expressed as the IC_50_—the concentration of HSs, at which the concentration of ABTS decreased by 50%.

The antiradical activity of the sample containing the HS sample was investigated by colorimetry using the stable chromogenic radical 2,2-diphenyl-1-picrylhydrazyl (DPPH) on a PE-5400 UV spectrophotometer (Ecroskhim, St. Petersburg, Russia).

The rate constant for the reaction occurring between most natural antioxidants and DPPH in protophilic solvents (methanol, ethanol) has values less than 1 L/mol*s; therefore, the reaction was carried out directly in cuvettes already placed in the device compartment by adding a DPPH solution to the HS sample (room temperature) [74]. The composition of the analyzed sample included 0.1 mL of a 0.001% DPPH solution (solvent–methanol) and 0.9 mL of the HS sample at final concentrations of 10; 25; 50; 75; 100; 125; 150 µg/mL. A mixture containing 0.9 mL of the test methanol solution of the HS sample and 0.1 mL of methanol served as the reference solution. The control sample consisted of 0.9 mL of methanol and 0.1 mL of a 0.001% methanol DPPH solution (methanol–comparison solution).

A reduction in the absorption of the analyzed solution indicated the radical-binding activity of the HS sample, which was as the IC_50_—the concentration of HSs, at which the concentration of DPPH decreased by 50% [74]. 

#### 3.7.2. Ability to Inhibit the Superoxide Anion Radical

The interaction of the HS sample with the superoxide anion radical (O_2_^−^•) was investigated by directly measuring its concentration in the system. To maintain a constant level of O_2_^−^•, a non-enzymatic system was used for its generation. In the non-enzymatic generation of O_2_^−^•, an electron is transferred from NADH to O_2_ via phenazine methosulfate (PMS), resulting in the formation of O_2_^−^•, which reduces nitro blue tetrazolium (NBT) to formazan with an absorption maximum at 560 nm. The incubation mixture with a total volume of 1 mL contained 20 mM KH_2_PO_4_–KOH buffer (pH 7.4), 6 µM PMS, 75 µM NADH, and 50 µM NBT. The HS sample was introduced into the model system in the form of an aqueous solution, achieving final concentrations of HSs: 2.5; 5; 10; 50; and 100 µg/mL. The absorption of the sample was measured on an SF-2000 spectrophotometer (Spectr, St. Petersburg, Russia) at a wavelength of 560 nm. The ability of the HS to suppress the O_2_^−^•-dependent reduction in NBT was assessed by the inhibition efficiency of this reaction (in %). Antioxidant activity was evaluated based on the IC_50_ value—the concentration of the HS sample at which the rate of NBT reduction reaction decreased by 50%. Water-soluble antioxidant ascorbic acid (AA) was used as a positive control [75,76].

#### 3.7.3. Cathodic Voltammetry

The antioxidant activity of the HS sample was determined by the method of cathodic voltammetry using the “AOA” analyzer (Tomsk) employing a mercury film electrode, according to the procedure described in [44,45]. As comparison drugs, ascorbic acid and the flavonoid dihydroquercetin (USP Reference Standard) were used. Solutions of HSs with a concentration of 0.0001 g/mL were prepared. An aliquot of the HS sample solution (0.1 mL) was added to the cell containing the background solution (10 mL), resulting in an analyzed HS solution concentration of 1 × 10^−^⁶ g/mL. Measurement conditions included a constant-current mode of cathodic voltammetry, a potential sweep rate (W) of 30 mV/s, a working potential range from 0.0 to −0.7 V, solution stirring time of 20 s, and a resting time of 10 s. The indicator of antioxidant activity was the coefficient of catalytic activity (K, µmol/L*min), representing the degree of change in the O_2_ electrochemical reduction current (EO_2_ process) and characterizing the reducing ability of the HS [44,45].

#### 3.7.4. Chelating Activity

The specific iron-binding (chelating) activity was determined based on the reaction with the ferrozine–Fe^2+^ complex, according to the procedure described in the methodology [75]. The specific iron-binding (chelating) activity was determined by the reaction with the ferrozine–Fe^2+^ complex. The model system with a volume of 1 mL contained 0.15 M NaCl, 50 µM Fe^2+^, and 300 µM ferrozine. The HS sample was used at final concentrations of 12.5; 25; 50; 75; 100 µg/mL. Absorption was measured on the SF2000 spectrophotometer (Spectr, St. Petersburg, Russia) at a wavelength of 562 nm against a reference solution containing the HS sample at the corresponding final concentrations. The iron-binding activity was assessed based on the IC_50_ value—the concentration of the HS sample at which the color intensity of the ferrozine–Fe^2+^ complex in the model system was reduced by 50%. As a standard, a classical chelator was used—ethylenediaminetetraacetic acid (EDTA) (Sigma Aldrich, St. Louis, MO, USA).

#### 3.7.5. Ability to Inhibit Hydroxyl Radicals

The ability of the HS sample to interact with HO• produced in the Haber–Weiss reaction with deoxyribose was tested in a model system with and without ethylenediaminetetraacetic acid (EDTA). Generated malondialdehyde (MDA) was identified by reacting with thiobarbituric acid (TBA), which forms a colored complex at 532 nm wavelength under high temperature and acidic conditions [48]. Given the high chelating activity of the HS sample, it is necessary to take into account the possibility of binding Fe^3+^ with HS molecules, which can lead to a decrease in the concentration of hydroxyl radicals. It is known that EDTA binds Fe^3+^ ions into a complex capable of generating HO• [75]. Therefore, the ability of HSs to bind HO• was studied in a model system with and without EDTA. The samples contained 20 mM KH_2_PO_4_–KOH buffer (pH 7.4), 0.1 mM ascorbic acid, 2.8 mM deoxyribose, 1 mM H_2_O_2_, and either 0.1 mM FeCl_3_ or 0.1 mM FeCl_3_ and 1 mM EDTA, pre-mixed in equal volumes. The experimental samples were supplemented with HS solutions at final concentrations of 0.5; 1; 1.5; 2 mg/mL. The control and experimental samples were incubated for 1 h at 37 °C; thereafter, 1 mL of 0.5% TBA and 1 mL of 10% trichloroacetic acid (TCA) were added to 0.5 mL of the reaction medium, followed by boiling in a water bath for 15 min, cooling, centrifuging, and measuring the optical density of the supernatant on the SF2000 spectrophotometer (Spectr, St. Petersburg, Russia) at a wavelength of 532 nm. Based on the dose-response curve, the concentration of the coal HS sample was calculated, at which there was 50% inhibition of MDA formation from deoxyribose under the action of HO•. Mannitol, a classical scavenger of hydroxyl radicals, was used as a reference substance.

Also, the potential binding of Fe^3+^ ions with HS molecules, which may result in a reduction in hydroxyl radical concentrations, was studied. EDTA forms complexes with Fe3+ ions capable of generating HO• [75]. Hence, the investigation focused on assessing the ability of HSs to bind HO• in a model system with and without EDTA. The experimental model system comprised 20 mM KH2PO4–KOH buffer (pH 7.4), 0.1 mM ascorbic acid, 2.8 mM deoxyribose, 1 mM H_2_O_2_, and either 0.1 mM FeCl_3_ or 0.1 mM FeCl3 along with 1 mM EDTA, pre-mixed in equal volumes. Various concentrations of HS solutions (0.5; 1; 1.5; 2 mg/mL) were introduced to the samples. Subsequently, the control and experimental samples were incubated for one hour at 37 °C. Following incubation, 0.5 mL of the reaction medium was combined with 1 mL of 0.5% TBA and 1 mL of 10% trichloroacetic acid (TCA). Then, samples were subjected to a boiling water bath for 15 min, followed by cooling, centrifugation, and measurement of the absorption of the supernatant at a wavelength of 532 nm using the SF2000 spectrophotometer (Spectr, St. Petersburg, Russia). The concentration of the HS sample required to inhibit MDA formation from deoxyribose by 50% was determined based on the dose-response curve. Mannitol served as a standard reference substance in the study.

#### 3.7.6. In Vitro Study of Antioxidant Activity

The intracellular production of reactive oxygen species was assessed with the dichlorodihydrofluorescein diacetate (DCFDA) method according to a previously established protocol [77]. The induction of intracellular reactive oxygen species production was carried out using three prooxidants—hydrogen peroxide (H_2_O_2_), tert-butyl hydroperoxide (t-BHP) and ferrous sulfate (FeSO_4_)—which exert their effects through different mechanisms.

HepG2 cells were seeded onto black 96-well culture plates (1 × 10⁴ cells per well). Subsequently, the cells were treated with the HS sample (25 µg/mL) and incubated for 24 h. The final concentration of HSs (25 µg/mL) in this study was chosen based on the results of cytotoxicity investigation. At the selected dose, the test sample did not affect cell viability. The results from our previous studies of other humic objects were also taken into account. After incubation, the cells were washed to remove HSs and treated with 10 µM working solution of DCFDA, followed by incubation in a thermostat for 20 min at 37 °C. After washing off the DCFDA, prooxidants were added at final concentrations of 100 µM H_2_O_2_, 25 µM t-BHP, or 10 µM FeSO_4_. Following incubation for 60 min at 37 °C, fluorescence was measured in the wells with excitation at λ_ex_ = 485 nm and emission at λ_em_ = 530 nm, and reactive oxygen species’ concentration was expressed in fluorescence units.

### 3.8. Immunotropic Activity

In the immunotropic activity study, C57BL/6 female mice (*n* = 20) aged 8–10 weeks were used. Animals were obtained from the Department of Experimental Biological Models at the E.D. Goldberg Research Institute for Pharmacology and Regenerative Medicine (Tomsk, Russia)—protocol number 171052020 of the Bioethical Committee dated 18 May 2020.

Peritoneal macrophages were harvested by lavaging the mouse peritoneal cavity with ice-cold physiological saline. Peritoneal macrophages from the resulting cell suspension were isolated using the EasySep™ Biotin Positive Selection Kit alongside the Anti-Mouse F4/80 Antibody (Stem Cell Technologies, Vancouver, BC, USA), following magnetic separation according to the provided protocol. Macrophages were then transferred to flat-bottomed 96-well plates (2.5–3 million/mL per well) and cultured under specified conditions in the presence of the HS sample (10 μg/mL) or lipopolysaccharide mitogen (LPS, 0.1 μg/mL). After 24 h of incubation, supernatants were collected from the wells and the concentration of pro-inflammatory cytokines (TNF-α, IL-1β) was measured by solid-phase enzyme-linked immunosorbent assay (ELISA) using commercial test systems (STEMCELL Technologies, Invitrogen, Seattle, WA, USA) according to the accompanying protocols [78,79]. 

After 48 h of incubation, nitrogen oxide (II) production was assessed based on the nitrite content in the supernatant and arginase activity. Nitrite levels in the cell supernatants were determined by the Griess reagent method (Sigma-Aldrich, St. Louis, MO, USA). Post-incubation, macrophage supernatants were collected from the wells, mixed with an equal volume of reagent, and the nitrite concentration was measured using a ChemWell 2910 analyzer (Awareness Technology, Palm City, FL, USA) at a wavelength of 540 nm. A standard curve with sodium nitrite solutions was used to determine nitrite concentration [80,81,82]. Arginase activity was assessed in the macrophages’ lysates using the modified method [83]. After 48 h of incubation with the HS sample, the supernatants were removed, cells were lysed with 0.1 mL of 0.1% Triton X-100 solution (Sigma Aldrich, St. Louis, MO, USA), and incubated for 10 min with shaking at room temperature. For arginase activation, 0.1 mL of 25 mM Tris-HCl solution (Sigma, USA) and 0.05 mL of 10 mM manganese chloride solution (ReaChem, Staraya Kupavna, Moscow region, Russia) were added to the wells and incubated at 56 °C for 10 min. Thereafter, 0.05 mL of the lysate was mixed with 0.05 mL of 0.5 M L arginine solution (Sigma Aldrich, St. Louis, MO, USA, pH 9.7) and incubated at 37 °C for 60 min. The reaction was halted by adding 0.4 mL of a concentrated sulfuric and phosphoric acid mixed with water (in a 1/3/7 volume ratio). The urea concentration in the resulting solution was determined using the Urea-450 test system (Erba Lachema, Karásek, Czech Republic) according to the protocol provided with the test system, applying the ChemWell 2910 analyzer (Awareness Technology, Palm City, FL, USA) at a wavelength of 540 nm. One unit of enzyme activity is defined as the amount of arginase that catalyzes the formation of 1 μM urea per minute [84,85].

Splenocytes were obtained by homogenizing spleens and washing them twice with physiological saline. Spleens were excised, homogenized in a glass homogenizer, filtered through a four-layer nylon mesh, washed twice with cold physiological saline, and resuspended in culture medium. Splenocytes were cultured in round-bottomed 96-well plates (2.5–3 million/mL per well) under the above-mentioned conditions in the presence of the mitogen Concanavalin A (ConA, 4 μg/mL) and coal HS sample (50 μg/mL). After 24 h, the supernatant was collected and the IL-2 content was measured by a solid-phase enzyme-linked immunosorbent assay using commercial test systems (eBioscience, San Diego, CA, USA), following the accompanying protocols.

### 3.9. Biological Activity

The use of animals in research of the biological activity and toxicity assessment of the HS sample has been approved by the Ethical Committee of the Siberian State Medical University under the Ministry of Health of Russia (protocol number 8199 dated 27 March 2020). The study was conducted on female Balb/C mice (for assessment of actoprotective activity) and CD-1 mice (for nootropic activity evaluation). Animals were obtained from the Research Institute of Pharmacology and Regenerative Medicine (Tomsk, Russia). The care and maintenance of animals were carried out in accordance with the standard operating procedures of the preclinical research center of the central scientific laboratory of the Siberian State Medical University (Tomsk, Russia).

Meldonium (Grindex, Rīga, Slovakia) and Piracetam (Renovation, Novosibirsk, Russia) were used as reference drugs. The doses of the reference drugs were calculated using human-to-mouse interspecies dose transfer factors [86]. Piracetam was administered at a dose of 300 mg/kg, while Meldonium was administered at a dose of 200 mg/kg. These doses correspond to literature data for Piracetam [87] and Meldonium [88].

The effective dose for the HS sample was selected during a normobaric hypercapnic hypoxia test. Based on the results of a 28-day toxicity study, HSs at the effective dose (500 mg/kg) had no toxic effects.

To evaluate the actoprotective properties of the studied sample, a 10-day course of intragastric administration of the HS sample at a dosage of 500 mg/kg or reference drug Meldonium (200 mg/kg) was performed. One hour after the last administration, animals with a load constituting 5% of their body weight were placed in a pool with a water temperature of 25 °C. The endurance of each mouse was measured as the swimming time until exhaustion, determined by observing uncoordinated movements and the inability to resurface within 7 s [89]. Thereafter, the animal was removed from the water, euthanized, and intracardiac blood sampling was carried out; the blood serum was assessed for lactate levels (“Lactate-Novo” kit, Vector-Best, Novosibirsk, Russia) [90].

The assessment of nootropic activity was conducted by evaluating the acquisition of the conditioned reflex of passive avoidance (CRPA), modified to a step-down paradigm, using the “Shelter” hardware–software complex (Neurobotics Trading LLC, Moscow, Zelenograd, Russia) [91]. A single intragastric dose of the investigated HS sample was administered to mice at a dose of 500 mg/kg. Fifteen minutes after the administration of the HS sample or reference drug Piracetam (300 mg/kg), the animals in the experimental groups were intraperitoneally administered with scopolamine (an amnestic agent inducing cholinergic dysfunction in the central nervous system) at a dose of 1 mg/kg (Sigma Aldrich, St. Louis, MO, USA); animals in the control group received an equivalent volume of the physiological solution (0.3 mL) intraperitoneally [68]. Twenty minutes later, for the acquisition of CRPA, the animal was placed on a plastic platform located on the electrode floor in the center of the chamber within the hardware–software complex. When the animal stepped down from the platform, an electric stimulation was triggered (current strength of 4 mA). The time the animals remained on the platform was recorded. The training session was repeated two more times. Two hours after the third training session, the animal was again placed on the platform, and the time spent on it was recorded for 60 s. The criterion for successful acquisition of CRPA was the animal remaining on the platform for the full 60 s.

### 3.10. Toxicity Assessment In Vivo

Acute and repeated dose toxicity of the HS sample was evaluated in Wistar rats, while allergenic potential was assessed in guinea pigs. The animals were obtained from the Research Institute of Pharmacology and Regenerative Medicine in Tomsk and housed in a controlled barrier facility.

For the acute toxicity assessment (*n* = 3), a single dose of HS 2000 mg/kg was administered intragastrically. The volume of administration was 0.8 ± 0.1 mL. Animals of the control group *(n* = 3) received an equal volume of physiological solution. Clinical monitoring of the animals was continuous for the hours following administration, followed by bi-daily observations for a duration of 14 days. During this period, mortality, toxicological symptoms, and the general health status of the animals were documented. All animals underwent necropsy at the end of the observation period [92]. All observations were compared with a control group of animals.

For the repeated dose toxicity assessment, the HS sample at a dose of 1000 mg/kg was administrated intragastrically to experimental rats (*n* = 15) once daily for 28 days. Animals of the control group (*n* = 10) received an equal volume of physiological solution. Animals of the control and experimental groups underwent daily visual inspections. Body weight measurements were recorded prior to the first administration and then weekly on days 8, 15, 22, 29, 36, and 43. Consumption of food and water was also recorded weekly on the same days. Hematological parameters were determined in rats by collecting 0.5 mL of blood from the heart post-mortem on days 29 (*n* = 10) and 43 (*n* = 5), after CO_2_ euthanasia, into capillary blood tubes containing K3-EDTA for analysis using a MindRay BC-2800Vet hematology analyzer (Shenzhen Mindray Bio-Medical Electronics Co., Ltd., Shenzhen, China). Biochemical parameters in rats were assessed on days 29 and 43 by collecting 5–6 mL of blood post-mortem from the heart with a 21G needle syringe. Analysis was conducted using an Architect automatic biochemistry analyzer (Abbott Laboratories, Chicago, IL, USA). Renal metabolism markers were assessed on days 28 and 42 using UrineRS test strips (High Technology Inc., North Attleboro, MA, USA). Urine collection was performed in individual metabolic cages (‘3 W’, China) where the rats resided for a 4 h period.

Complete necropsies were performed immediately following euthanasia. Organ weight coefficients were calculated for each organ. For histological analysis, tissue samples were taken from three animals per group for the following organs: brain, heart, lung, liver, kidney, urinary bladder, stomach, small and large intestine, pancreas, thyroid, spleen, thymus, adrenal gland, ovary and uterus or testis and prostate gland. The tissues were fixed in a 10% buffered formalin solution and underwent routine processing followed by paraffin embedding. From the paraffin blocks, sections were cut and stained with hematoxylin and eosin. Histopathological assessments of the slides were conducted using light microscopy, including evaluation and description of the condition of the examined specimens.

### 3.11. Allergenicity Assessment

The sensitizing effect of the HS sample was investigated through a regime of single daily epicutaneous applications to 2 × 2 cm areas on the lateral surfaces of male and female guinea pigs’ bodies, five times per week, resulting in a total of 20 applications, as described in [93] with minor modifications. The study included the following animal groups: control male guinea pigs (*n* = 5), male guinea pigs treated with coal HS sample 125 mg/mL (*n* = 5), control female guinea pigs (*n* = 5), and female guinea pigs treated with coal HS sample 125 mg/mL (*n* = 5). Animals of the control groups received percutaneous applications of physiological solution. The initial test was conducted 20 min after the 10th application, and a subsequent test followed the 20th application. Skin reactions were assessed according to the following criteria: if the skin in the application area remained unchanged, the reaction was considered negative (−); a weak positive reaction (+) was indicated by the presence of erythema at the site of application; the presence of both erythema and skin edema was evaluated as a positive reaction (++); a strong positive reaction (+++) was characterized by erythema and edema extending beyond the area of contact with the humic substance solution; and the highest degree of reaction (++++), hyperergy, was evidenced by extensive peripheral edema, erythema, vesiculation, and skin ulceration.

### 3.12. Statistical Analysis

In all experiments, the mean and the standard error (X ± SE) were calculated. Except for in vivo toxicity assessments and actoprotective and nootropic activity studies, the acquired data were statistically processed using Statistica 8.0 software (StatSoft, Inc., Tulsa, OK, USA), with Shapiro–Wilk’s test applied to verify the normality of distribution. Intergroup comparisons were made using Dunnett’s test for comparing multiple experimental groups with one control group. For single and repeated dose toxicity studies, statistical analysis was performed using analysis of variance (ANOVA) with Benjamini–Hochberg correction for multiple comparisons (software SPSS 17.0). For in vivo studies of biological activity, Kruskal–Wallis criteria was employed for statistical processing (Statistica 8.0 software). In all experiments, differences between groups were considered statistically significant at *p* < 0.05.

## 4. Conclusions

The objective of the current study was to investigate the chemical structure descriptors and biomedical properties of HSs from brown coal. The sample analyzed in the study was identified as coal-derived HSs based on the detection of key molecular structural parameters typically associated with this type of natural compound. An evaluation of chemical, microbiological, and pharmacological safety parameters of this new food substance was conducted, as well as the effectiveness of its biological application. It was determined that the studied HS sample met all the necessary safety requirements, including microbiological purity and content of heavy metals and radionuclides. 

The HS samples were proven to lack general and systemic toxicity, cytotoxicity, and allergenic properties. In vivo experiments showed that a single intragastric administration of an HS was well-tolerated by animals, with no mortality or clinical signs of health disturbances observed. Necropsy analysis revealed no pathomorphological changes in organs. Furthermore, a 28-day repeated intragastric dosing of the HS did not result in mortality, changes in clinical status, or alterations in hematological and biochemical parameters compared to control values. Macroscopic and histological examination of organs and tissues also showed no abnormalities. Additionally, a 20-day topical application of HSs did not lead to sensitization of animals in the skin application test. These findings support the safety and potential therapeutic benefits of HSs for further development as a dietary supplement. 

The results demonstrate that the HS sample exhibits high antioxidant and antiradical activity, as well as immunotropic and cytoprotective properties. Pronounced actoprotective and nootropic activities of the studied HS sample were demonstrated. A single intragastric administration led to the restoration of the ability of animals with scopolamine-induced amnesia to form conditioned reflexes at control levels, suggesting that the nootropic effect of the HS may be mediated through its influence on the cholinergic system.

Considering all this information, it can be concluded that the HSs isolated from the Kansk-Achinsk coal basin (Krasnoyarsk Territory, Russia) are safe and effective natural substances for use as potential pharmaceutical compounds and as dietary bioactive supplements.

## Figures and Tables

**Table 1:** Elemental (C, H, N, O) composition of the studied HS sample: mass/atomic fractions of elements (%) and atomic ratios (H/C, O/C, N/C) (*n* = 3).

Mass Fractions of Elements, % Atomic Fractions of Elements, %				Atomic Ratios		
C	H	N	O	H/C	O/C	N/C
44.9 ± 0.48 32.9 ± 0.43	4.51 ± 0.040 39.28 ± 0.30	1.09 ± 0.01 0.70 ± 0.01	49.48 ± 0.42 27.15 ± 0.16	1.20	0.83	0.02

**Table 2:** Results of the studied HS sample antioxidant activity (AOA) study (*n* = 3).

Free Radical/Fe^2+^	IC_50_, µg/mL	
	Coal HS Sample	Reference Compounds
DPPH	27.3 ± 0.2	21.2 ± 0.4 ^1^
ABTS•^+^	10.8 ± 0.3	3.4 ± 0.2 ^2^
O_2_^−^•	20.5 ± 1.7	13.3 ± 0.9 ^3^
Fe^2+^	25.7 ± 0.5	9.6 ± 0.2 ^4^
HO•	2.4 ± 0.2 mg/mL	0.9 ± 0.0 mg/mL ^5^

Note: ^1^—dihydroquercetin, ^2^—trolox, ^3^—ascorbic acid, ^4^—EDTA, ^5^—mannitol.

**Table 3:** Results of determining the catalytic antioxidant activity of the studied HS sample using voltametric method (*n* = 3).

Studied Sample/Reference Substances	K, µmol/L*min
Coal HS	0.91 ± 0.08
Ascorbic acid	1.15 ± 0.10
Dihydroquercetin	0.78 ± 0.08

**Table 4:** Results of the evaluation of the actoprotective properties of the studied HS sample in the “swimming exhaustion” test (X ± SE).

Experimental Groups	Swimming Time, min	*p*-Value
Control (*n* = 10)	19.5 ± 3.4	—
Meldonium, 200 mg/kg (*n* = 10)	37.5 ± 9.9	*p*_2-1_ = 0.36
HS, 500 mg/kg (*n* = 10)	34.7 ± 4.6	*p*_3-1_ = 0.04 *p*_3-2_ = 0.39

**Table 5:** Results of the assessment of lactate levels in the serum of mice following administration of the studied HS sample and the reference drug Meldonium (X ± SE).

Experimental Groups	Lactate Level, mmol/L	*p*-Value
Control (*n* = 10)	11.59 ± 0.93	—
Meldonium, 200 mg/kg (*n* = 10)	7.44 ± 0.54	*p*_2-1_ = 0.001
HS, 500 mg/kg (*n* = 10)	8.71 ± 0.46	*p*_3-1_ = 0.01 *p*_3-2_ = 0.09

**Table 6:** Evaluation of the effect of the studied HS sample or reference drug Piracetam on the development of CRPA in mice (X ± SE).

Experimental Groups	Time Spent on the Platform, Sec	*p*-Value
Control (*n* = 10)	40.50 ± 6.17	—
Scopolamine, 1 mg/kg (amnestic control) (*n* = 10)	13.20 ± 5.80	*p*_2-1_ = 0.005
Piracetam, 300 mg/kg + Scopolamine, 1 mg/kg (*n* = 10)	51.50 ± 4.89	*p*_3-1_ = 0.17 *p*_3-2_ = 0.001
HS, 500 mg/kg + Scopolamine, 1 mg/kg (*n* = 10)	37.00 ± 6.53	*p*_4-1_ = 0.70 *p*_4-2_ = 0.01

## Data Availability

Data are contained within the article and Supplementary Materials.

## Supplementary Materials

The following supporting information can be downloaded at: https://www.mdpi.com/article/10.3390/molecules29071530/s1. Table S1: Body weight (g) of female rats after single intragastric administration of the studied HS sample at a dose of 2000 mg/kg (X ± SE); Table S2: Body mass (g) of rats during a 28-day course administration of the studied HS sample at a dose of 1000 mg/kg body weight and subsequent post-cancellation period (X ± SE); Table S3: Mass of feed consumed (g) by rats during a course of 28-day administration of the studied HS sample at a dose of 1000 mg/kg, and subsequent post-cancellation period (X ± SE); Table S4: Volume of water consumption (mL) by rats during a course of 28-day administration of the studied HS sample at a dose of 1000 mg/kg, and subsequent post-cancellation period (X ± SE); Table S5: Parameters of clinical laboratory blood analysis in rats during a course of 28-day administration of the studied HS sample at a dose of 1000 mg/kg, and subsequent post-cancellation period (X ± SE); Table S6: Biochemical parameters of rat serum during a course of 28-day administration of the studied HS sample at a dose of 1000 mg/kg, and subsequent post-cancellation period (X ± SE); Table S7: Indicators of renal metabolism in rats during a course of 28-day administration of the studied HS sample at a dose of 1000 mg/kg, and subsequent post-cancellation period (X ± SE); Table S8: Mass of internal organs in male rats during a course of 28-day administration of the studied HS sample at a dose of 1000 mg/kg, and subsequent post-cancellation period (X ± SE); Table S9: Mass of internal organs in female rats during a course of 28-day administration of the studied HS sample at a dose of 1000 mg/kg, and subsequent post-cancellation period (X ± SE); Table S10: Assessment of the impact of dermal applications of the studied HS sample on the condition of the skin of guinea pigs.
