# Supplementary material for: Coal-Derived Humic Substances: Insight into Chemical Structure Parameters and Biomedical Properties
Source: Molecules. 2024 Mar 29;29(7):1530. doi: 10.3390/molecules29071530 (PMC11013056; doi:10.3390/molecules29071530)
Supplement: Supplementary file 1 [file molecules-29-01530-s001.zip › molecules-2876782-supplementary.pdf]

Table S1- Body weight (g) of female rats after single intragastric administration of the studied HS sample at a dose of 2000 mg/kg (X±SE)

| Experimental Groups | Day 1 (pre-administration) | Day 8 | Day 15 |
|---|---|---|---|
| Control (n=3) | 286,40±7,60 | 290,10±9,30 | 291,00±7,20 |
| Coal HS (n=3) | 291,30±4,80 | 294,00±4,40 | 300,50±3,30 |

Table S2 – Body mass (g) of rats during a 28-day course administration of the studied HS sample at a dose of 1000 mg/kg body weight and subsequent post-cancellation period (X±SE)

| Day | Control group (n=10) | Coal HS administration for 28 days (n=10) | Post-cancellation period (43 days) (n=5) |
|---|---|---|---|
| Male rats | | | |
| Day 1 (pre-administration) | 464,40±11,00 | 465,10±10,30 | 463,20±17,70 |
| Day 8 | 467,30±10,70 | 468,90±10,30 | 466,40±17,80 |
| Day 15 | 482,50±10,00 | 485,30±8,90 | 483,20±17,00 |
| Day 22 | 504,00±10,50 | 506,10±9,10 | 501,60±17,50 |
| Day 29 | 522,90±9,80 | 525,80±9,80 | 519,20±17,40 |
| Day 36 | - | - | 539,20±16,20 |
| Day 43 | - | - | 554,40±16,20 |
| Female rats | | | |
| Day 1 (pre-administration) | 260,50±4,20 | 264,00±4,10 | 263,00±6,30 |
| Day 8 | 263,10±4,10 | 266,90±4,00 | 266,00±5,60 |
| Day 15 | 267,70±4,20 | 270,80±3,60 | 270,00±5,80 |
| Day 22 | 277,30±4,60 | 281,20±3,40 | 280,80±6,80 |
| Day 29 | 284,60±4,10 | 289,40±3,60 | 286,80±6,50 |
| Day 36 | - | - | 297,20±5,40 |
| Day 43 | - | - | 306,60±7,30 |

Table S3 – Mass of feed consumed (g) by rats during a course of 28-day administration of the studied HS sample at a dose of 1000 mg/kg, and subsequent post-cancellation period (X±SE)

| Day | Control group (n=10) | Coal HS administration for 28 days (n=10) | Post-cancellation period (43 days) (n=5) |
|---|---|---|---|
| Male rats | | | |
| Day 8 | 21,30±0,90 | 20,30±1,10 | 22,10±0,90 |
| Day 15 | 20,10±1,00 | 21,30±1,10 | 19,80±1,50 |
| Day 22 | 22,00±0,90 | 23,40±1,09 | 21,30±1,80 |
| Day 29 | 23,20±0,80 | 21,90±0,60 | 22,90±1,30 |
| Day 36 | - | - | 24,40±1,90 |
| Day 42 | - | - | 24,10±1,90 |
| Female rats | | | |
| Day 8 | 40,5±3,6 | 39,3±2,7 | 39,8±4,1 |
| Day 15 | 39,3±2,7 | 40,9±3,4 | 39,0±6,3 |
| Day 22 | 41,5±2,9 | 42,0±3,4 | 42,2±5,2 |
| Day 29 | 41,9±4,7 | 40,9±5,8 | 39,6±4,1 |
| Day 36 | - | - | 41,0±5,8 |
| Day 42 | - | - | 39,2±7,2 |

Table S4 – Volume of water consumption (mL) by rats during a course of 28-day administration of the studied HS sample at a dose of 1000 mg/kg, and subsequent post-cancellation period (X±SE)

| Day | Control group (n=10) | Coal HS administration for 28 days (n=10) | Post-cancellation period (43 days) (n=5) |
|---|---|---|---|
| Male rats | | | |
| Day 8 | 17,40±1,90 | 19,10±1,00 | 18,60±1,30 |
| Day 15 | 17,20±0,60 | 17,40±0,50 | 18,10±0,60 |
| Day 22 | 16,80±0,80 | 16,10±0,50 | 17,50±0,30 |
| Day 29 | 17,30±0,70 | 16,70±0,50 | 17,40±0,80 |
| Day 36 | - | - | 17,80±0,60 |
| Day 42 | - | - | 18,50±1,40 |
| Female rats | | | |
| Day 8 | 35,5±3,6 | 35,9±1,2 | 34,8±4,1 |
| Day 15 | 38,1±3,9 | 38,7±6,2 | 39,4±2,1 |
| Day 22 | 39,1±4,2 | 38,6±3,5 | 37,8±2,3 |
| Day 29 | 36,8±2,4 | 36,6±3,5 | 38,4±2,1 |
| Day 36 | - | - | 37,8±2,1 |
| Day 42 | - | - | 35,6±1,2 |

Table S5 – Parameters of clinical laboratory blood analysis in rats during a course of 28-day administration of the studied HS sample at a dose of 1000 mg/kg, and subsequent post-cancellation period (X±SE)

| Indicator | | Control group (n=10) | Coal HS administration | |
|---|---|---|---|---|
| | | | For 29 days (n=10) | Post-cancellation period (43 days) (n=5) |
| Male rats | | | | |
| Hemoglobin, g/L | | 219,7±5,6 | 206,2±5,7 | 220,2±5,5 |
| Erythrocytes, $10^{12}$/L | | 9,8±0,2 | 9,5±0,2 | 9,7±0,2 |
| MCH (mean corpuscular hemoglobin), pg | | 22,3±0,2 | 23,8±1,2 | 22,8±0,4 |
| MCHC (mean corpuscular hemoglobin concentration), g/L | | 441,7±2,1 | 436,8±4,2 | 438,6±4,0 |
| Hematocrit, % | | 49,7±1,2 | 47,1±1,1 | 50,2±1,2 |
| Platelets, $10^9$/L | | 947,6±63,6 | 925,2±84,0 | 944,8±109,7 |
| Total leukocyte count, $10^9$/L | | 13,62±0,8 | 12,9±0,8 | 12,4±1,0 |
| Granulocytes | $10^9$/L | 3,6±0,5 | 4,0±0,6 | 3,5±0,9 |
| | % | 25,7±2,8 | 29,6±3,6 | 27,6±5,4 |
| Lymphocytes | $10^9$/L | 9,6±0,6 | 8,4±0,4 | 8,5±0,7 |
| | % | 70,8±2,8 | 66,5±3,7 | 69,5±5,6 |
| Monocytes | $10^9$/L | 0,47±0,03 | 0,50±0,04 | 0,38±0,08 |
| | % | 3,5±0,2 | 3,9±0,3 | 3,0±0,4 |
| Female rats | | | | |
| Hemoglobin, g/L | | 195,6±5,1 | 188,3±5,6 | 215,8±8,6 |
| Erythrocytes, $10^{12}$/L | | 8,4±0,2 | 8,8±0,2 | 8,4±1,0 |
| MCH (mean corpuscular hemoglobin), pg | | 23,2±0,1 | 21,5±1,0 | 23,5±0,3 |
| MCHC (mean corpuscular hemoglobin concentration), g/L | | 440,8±1,9 | 405,9±19,2 | 442,8±2,0 |
| Hematocrit, % | | 44,3±1,1 | 46,9±1,4 | 48,7±2,0 |
| Platelets, $10^9$/L | | 984,0±89,9 | 979,7±92,6 | 978,0±158,8 |
| Total leukocyte count, $10^9$/L | | 10,2±0,8 | 9,8±0,7 | 9,8±1,7 |
| Granulocytes | $10^9$/L | 2,5±0,4 | 2,6±0,5 | 2,7±0,6 |
| | % | 25,5±3,9 | 26,6±3,4 | 27,5±2,5 |
| Lymphocytes | $10^9$/L | 7,4±0,8 | 6,9±0,6 | 6,8±1,1 |
| | % | 74,9±4,0 | 70,8±3,5 | 69,8±2,6 |
| Monocytes | $10^9$/L | 0,24±0,04 | 0,24±0,03 | 0,26±0,04 |
| | % | 2,6±0,5 | 2,5±0,3 | 2,7±0,2 |

Table S6 – Biochemical parameters of rat serum during a course of 28-day administration of the studied HS sample at a dose of 1000 mg/kg, and subsequent post-cancellation period (X±SE)

| Indicator | Control group (n=10) | Coal HS administration | |
|---|---|---|---|
| | | For 29 days (n=10) | Post-cancellation period (43 days) (n=5) |
| Male rats | | | |
| ALT (Alanine Aminotransferase), U/L | 49,40±1,90 | 45,90±2,20 | 57,20±7,70 |
| AST (Aspartate Aminotransferase), U/L | 123,70±4,70 | 127,00±5,50 | 129,80±7,00 |
| ALP (Alkaline Phosphatase), U/L | 133,00±6,90 | 126,80±7,30 | 125,20±14,20 |
| Glucose, mmol/L | 4,80±0,23 | 5,30±0,25 | 4,38±0,21 |
| Urea, mmol/L | 4,69±0,18 | 4,37±0,12 | 4,92±0,43 |
| Creatinine, µmol/L | 46,26±1,80 | 48,22±2,62 | 49,38±1,54 |
| Total Protein, g/L | 70,90±1,40 | 70,20±1,50 | 72,80±1,90 |
| Albumin, g/L | 31,50±1,10 | 32,40±0,50 | 34,20±1,20 |
| Globulin, g/L | 39,40±1,80 | 37,80±1,50 | 38,60±2,50 |
| Albumin/Globulin Ratio | 0,82±0,06 | 0,87±0,05 | 0,90±0,07 |
| Cholesterol, mmol/L | 1,73±0,08 | 1,76±0,09 | 1,98±0,16 |
| Triglycerides, mmol/L | 0,87±1,33 | 0,89±0,18 | 0,88±0,16 |
| Total Bilirubin, µmol/L | 2,31±0,12 | 2,51±0,12 | 2,40±0,17 |
| Direct Bilirubin, µmol/L | 1,54±0,06 | 1,58±0,05 | 1,68±0,18 |
| Phosphorus, mmol/L | 2,64±0,09 | 2,63±0,04 | 2,50±0,08 |
| Sodium, mmol/L | 141,20±1,90 | 142,50±2,40 | 144,40±3,40 |
| Potassium, mmol/L | 6,16±0,16 | 6,50±0,14 | 5,94±0,17 |
| Calcium, mmol/L | 3,00±0,08 | 3,03±0,04 | 2,91±0,03 |
| Chloride, mmol/L | 96,50±1,60 | 99,30±1,20 | 99,80±2,60 |
| Female rats | | | |
| ALT (Alanine Aminotransferase), U/L | 62,20±5,50 | 53,30±2,90 | 52,40±1,70 |
| AST (Aspartate Aminotransferase), U/L | 125,00±10,60 | 132,80±9,20 | 136,60±12,40 |
| ALP (Alkaline Phosphatase), U/L | 126,00±11,00 | 120,80±4,80 | 118,60±11,00 |
| Glucose, mmol/L | 5,07±0,28 | 5,72±0,14 | 4,92±0,51 |
| Urea, mmol/L | 4,88±0,25 | 5,70±0,47 | 5,30±0,35 |
| Creatinine, µmol/L | 44,13±0,75 | 46,62±1,19 | 45,30±1,36 |
| Total Protein, g/L | 70,70±1,30 | 71,80±1,90 | 72,00±2,10 |
| Albumin, g/L | 33,80±0,50 | 34,80±1,20 | 34,20±2,30 |
| Globulin, g/L | 36,90±1,20 | 37,00±1,10 | 37,80±1,00 |
| Albumin/Globulin Ratio | 0,92±0,03 | 0,95±0,03 | 0,91±0,07 |
| Cholesterol, mmol/L | 1,97±0,10 | 2,05±0,12 | 1,92±0,06 |
| Triglycerides, mmol/L | 0,71±0,06 | 0,88±0,10 | 0,84±0,07 |
| Total Bilirubin, µmol/L | 2,60±0,11 | 2,77±0,11 | 2,84±0,17 |
| Direct Bilirubin, µmol/L | 1,72±0,08 | 1,68±0,08 | 1,70±0,19 |
| Phosphorus, mmol/L | 2,35±0,13 | 2,49±0,06 | 2,60±0,08 |
| Sodium, mmol/L | 137,80±1,40 | 140,50±4,20 | 139,60±5,00 |
| Potassium, mmol/L | 5,78±0,24 | 6,04±0,11 | 6,30±0,26 |
| Calcium, mmol/L | 2,76±0,03 | 2,88±0,10 | 2,84±0,07 |
| Chloride, mmol/L | 98,90±1,70 | 103,10±1,40 | 97,40±2,60 |

Table S7 – Indicators of renal metabolism in rats during a course of 28-day administration of the studied HS sample at a dose of 1000 mg/kg, and subsequent post-cancellation period (X±SE)

| Indicator | Control group (n=10) | Coal HS administration | |
|---|---|---|---|
| | | For 29 days (n=10) | Post-cancellation period (43 days) (n=5) |
| Male rats | | | |
| Daily volume, mL | 8,8±1,8 | 9,4±2,1 | 8,6±2,4 |
| Specific gravity | 1,0220±0,0011 | 1,0225±0,0011 | 1,0220±0,0012 |
| pH | 6,8±0,1 | 6,9±0,1 | 6,7±0,3 |
| Glucose, mg/dL | 0,0±0,0 | 0,0±0,0 | 0,0±0,0 |
| Protein, mg/dL | 0,060±0,025 | 0,030±0,020 | 0,030±0,030 |
| Occult blood, cells/μL | 0,0±0,0 | 0,0±0,0 | 0,0±0,0 |
| Bilirubin, μmol/L | 0,0±0,0 | 0,0±0,0 | 0,0±0,0 |
| Urobilinogen, μmol/L | 0,0±0,0 | 0,0±0,0 | 0,0±0,0 |
| Nitrites | 0,0±0,0 | 0,0±0,0 | 0,0±0,0 |
| Leukocytes, cells/μL | 0,0±0,0 | 0,0±0,0 | 0,0±0,0 |
| Ketones, mg/dL | 0,0±0,0 | 0,0±0,0 | 0,0±0,0 |
| Female rats | | | |
| Daily volume, mL | 7,3±1,2 | 6,8±1,0 | 7,6±2,0 |
| Specific gravity | 1,0225±0,0008 | 1,0220±0,0008 | 1,0210±0,0010 |
| pH | 6,5±0,1 | 6,8±0,1 | 6,9±0,2 |
| Glucose, mg/dL | 0,0±0,0 | 0,0±0,0 | 0,0±0,0 |
| Protein, mg/dL | 0,060±0,025 | 0,045±0,023 | 0,030±0,030 |
| Occult blood, cells/μL | 0,0±0,0 | 0,0±0,0 | 0,0±0,0 |
| Bilirubin, μmol/L | 0,0±0,0 | 0,0±0,0 | 0,0±0,0 |
| Urobilinogen, μmol/L | 0,0±0,0 | 0,0±0,0 | 0,0±0,0 |
| Nitrites | 0,0±0,0 | 0,0±0,0 | 0,0±0,0 |
| Leukocytes, cells/μL | 0,0±0,0 | 0,0±0,0 | 0,0±0,0 |
| Ketones, mg/dL | 0,0±0,0 | 0,0±0,0 | 0,0±0,0 |

Table S8 – Mass of internal organs in male rats during a course of 28-day administration of the studied HS sample at a dose of 1000 mg/kg, and subsequent post-cancellation period (X±SE)

| Organ | Control group (n=10) | Coal HS administration | |
|---|---|---|---|
| | | For 29 days (n=10) | Post-cancellation period (43 days) (n=5) |
| Absolute mass, g | | | |
| Liver | 13,98±0,42 | 14,25±0,35 | 15,31±0,46 |
| Kidneys | 3,16±0,05 | 3,26±0,06 | 3,37±0,20 |
| Testes | 2,50±0,13 | 4,43±0,05 | 4,44±0,14 |
| Lungs | 2,23±0,10 | 2,25±0,08 | 2,50±0,13 |
| Brain | 1,96±0,07 | 2,04±0,03 | 2,25±0,12 |
| Heart | 1,51±0,05 | 1,60±0,04 | 1,64±0,06 |
| Spleen | 0,951±0,052 | 1,001±0,055 | 1,113±0,104 |
| Thymus | 0,510±0,038 | 0,489±0,028 | 0,558±0,051 |
| Adrenal glands | 0,090±0,005 | 0,093±0,003 | 0,100±0,005 |
| Total body mass | 522,90±9,80 | 525,80±9,80 | 554,40±16,20 |
| Relative mass, % | | | |
| Liver | 3,02±0,06 | 2,93±0,05 | 2,77±0,12 |
| Kidneys | 0,68±0,02 | 0,67±0,01 | 0,61±0,03 |
| Testes | 0,95±0,04 | 0,91±0,01 | 0,81±0,05 |
| Lungs | 0,48±0,02 | 0,46±0,02 | 0,45±0,02 |
| Brain | 0,42±0,01 | 0,42±0,01 | 0,40±0,01 |
| Heart | 0,33±0,01 | 0,33±0,00 | 0,30±0,02 |
| Spleen | 0,20±0,01 | 0,21±0,01 | 0,20±0,02 |
| Thymus | 0,11±0,01 | 0,10±0,01 | 0,10±0,01 |
| Adrenal glands | 0,02±0,00 | 0,02±0,00 | 0,02±0,00 |

Table S9 – Mass of internal organs in female rats during a course of 28-day administration of the studied HS sample at a dose of 1000 mg/kg, and subsequent post-cancellation period (X±SE)

| Organ | Control group (n=10) | Coal HS administration | |
|---|---|---|---|
| | | For 29 days (n=10) | Post-cancellation period (43 days) (n=5) |
| Absolute mass, g | | | |
| Liver | 8,91±0,36 | 9,26±0,35 | 10,12±0,27 |
| Kidneys | 1,87±0,05 | 1,90±0,06 | 2,05±0,14 |
| Brain | 1,78±0,05 | 1,75±0,06 | 1,89±0,04 |
| Lungs | 1,56±0,08 | 1,59±0,07 | 1,76±0,11 |
| Heart | 0,93±0,03 | 0,96±0,04 | 1,01±0,03 |
| Spleen | 0,684±0,074 | 0,725±0,025 | 0,921±0,129 |
| Thymus | 0,432±0,023 | 0,443±0,024 | 0,488±0,048 |
| Ovaries | 0,134±0,014 | 0,143±0,024 | 0,164±0,018 |
| Adrenal glands | 0,078±0,006 | 0,074±0,003 | 0,081±0,005 |
| Total body mass | 284,60±4,10 | 289,40±3,60 | 306,60±7,30 |
| Relative mass, % | | | |
| Liver | 3,34±0,10 | 3,43±0,081 | 3,30±0,05 |
| Kidneys | 0,71±0,01 | 0,72±0,01 | 0,67±0,05 |
| Brain | 0,68±0,02 | 0,66±0,03 | 0,62±0,01 |
| Lungs | 0,60±0,03 | 0,60±0,02 | 0,57±0,03 |
| Heart | 0,35±0,01 | 0,36±0,01 | 0,33±0,01 |
| Spleen | 0,26±0,03 | 0,27±0,01 | 0,30±0,05 |
| Thymus | 0,16±0,01 | 0,17±0,01 | 0,16±0,01 |
| Ovaries | 0,05±0,00 | 0,05±0,01 | 0,05±0,01 |
| Adrenal glands | 0,03±0,00 | 0,03±0,00 | 0,03±0,00 |

Table S10 - Assessment of the impact of dermal applications of the studied HS sample on the condition of the skin of guinea pigs

| Experimental Groups | Number of dermal applications | |
|---|---|---|
| | 10 | 20 |
| Control, males (n=5) | (-) | (-) |
| Humic substances 125 mg/ml, males (n=5) | (-) | (-) |
| Control, females (n=5) | (-) | (-) |
| Humic substances 125 mg/ml, females (n=5) | (-) | (-) |
